# Transcriptome, glycome, and mucinome analysis reveal zinc is essential for the composition of mucus in the human goblet cell model HT-29-MTX

**DOI:** 10.1038/s41598-026-66227-4

**Published:** 2026-08-11

**Authors:** Christoph Schüßler, Nara Chung, Renaud Léonard, Heike Sprenger, Tari Rödel, Keira E. Mahoney, Susanne Thomsen, Esther Ocket, Claudia Matthaeus, Jordan Denis, Franziska Ebert, Marvin Wolf, Willy Morelle, François Foulquier, Albert Braeuning, Catherine Robbe Masselot, Stacy A. Malaker, Maria Maares

**Affiliations:** 1 Department of Food Chemistry, Institute of Nutritional Science, University of Potsdam, Karl-Liebknecht-Str. 24–25, 14476 Potsdam-Golm, Germany; 2 Department of Chemistry, Yale University, New Haven, CT USA; 3 Université de Lille, CNRS, UMR 8576 - UGSF, Lille, France; 4 Department Chemical and Product Safety, German Federal Institute for Risk Assessment (BfR), Berlin, Germany; 5 Department of Food Chemistry and Toxicology, Technische Universität Berlin, Berlin, Germany; 6 Department of Cellular Physiology of Nutrition, Institute of Nutritional Science, University of Potsdam, Potsdam-Golm, Germany

**Keywords:** Glycosylation, Intestinal mucins, Trace elements, Zinc deficiency, Biochemistry, Diseases, Microbiology, Molecular biology

## Abstract

Zinc (Zn) deficiency affects approximately 1 billion people worldwide with severe consequences for their health, including increased intestinal infections, inflammation, and diarrhea. Accordingly, the intestinal defense barrier is compromised, leading to epithelial destruction and alteration of mucus. However, the processes and the extent to which Zn deficiency affects mucin synthesis in intestinal goblet cells (GCs) remain poorly understood. To this end, we investigated the impact of Zn deficiency on mucin expression and glycosylation in the human GC model HT-29-MTX. Zn deprivation altered the GC transcriptome, affecting genes involved in Zn transport, mucin synthesis and glycosylation. Accordingly, mucus composition was changed in Zn-deficient GCs, significantly increasing MUC2 and MUC17 on the mRNA and protein level. Several Zn transporters, mostly those associated with the early secretory pathway (ESP), were dysregulated, indicating an adaptive response of cellular Zn homeostasis. Additionally, free Zn was markedly reduced in the ESP, a critical location for glycosylation. Zn deficit substantially changed mucin glycosylation, characterized by an increase in sialylation and a strong decrease in complex *N*-glycans. All these changes involved widespread dysregulation of glycosyltransferase expression, including an increase in COSMC, a Zn-binding chaperone essential for the core 1 *O*-glycan formation. Collectively, our in vitro findings demonstrate that Zn is a critical regulator of mucin production and glycosylation in GCs. Zn deficiency might weaken the protective and functional qualities of intestinal mucus, increasing the risk of infections and potentially disrupting host-microbiome interactions.

**Supplementary Information:**

The online version contains supplementary material available at 10.1038/s41598-026-66227-4.

## Introduction

The trace element (TE) zinc (Zn) is essential for the catalytic, structural, and regulatory function of proteins and therefore is crucial for numerous physiological processes, such as the immune system and metabolism. As there is no Zn reservoir in the human body, it must be ingested daily to compensate for endogenous losses^1^. Zn deficiency is one of the most prevalent micronutrient deficiencies, affecting approximately 17% of the global population, thereby representing a significant health and economic burden worldwide^2^. The primary cause of Zn deficiency is either insufficient oral Zn intake or diets with low Zn bioavailability in the intestine, where Zn absorption via specific Zn/iron-regulated transporter-like proteins (ZIPs) and Zn transporters (ZnTs) occurs^3^. Symptoms of Zn deficiency encompass immune dysfunction, increased incidence of infection, slow wound healing, and metabolic disturbances. In addition, Zn deficiency significantly impacts gastrointestinal health, including altered intestinal homeostasis, increasing the likelihood of infection and, in severe cases, diarrhea^2^. The underlying (cellular) processes in the intestine leading to these symptoms, however, remain to be fully understood.

**Fig. 1:**
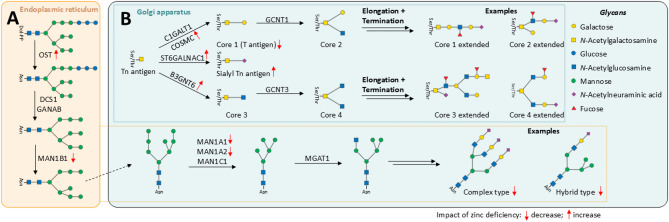
Schematic illustration of the ***N***- and ***O***-glycan biosynthesis in the secretory pathway and the impact of Zn deficiency. (**A**) *N*-glycans are formed through a multi-step process. High Man structures linked to dolichoyl-pyrophosphate (DOL-PP) are transferred via the oligosaccharyltransferase (OST) protein complex to Asn residues. These high Man structures are subsequently processed by cleaving, the protein undergoes folding and is transported to the Golgi, where *N*-glycans are further modified to generate high-Man complex type and hybrid type structures^10^. (**B**) Tn antigen (Ser/Thr residues linked with GalNAc) are elongated with Gal or GlcNAc, catalyzed by C1GALT1 or B3GNT6, respectively, which can lead to the formation of the core 1 or 3, respectively. These core *O*-glycans can be further processed to core 2 or 4 by glucosaminyl (*N*-acetyl) transferase (GCNT)1 or GCNT3, respectively. Core structures can then be further elongated^8^ or terminated by Neu5Ac (sialic acid), Fuc, Man, or sulfate^4,8^. Glycan structures were drawn with GlycoGlyph^14^. Red arrows show where Zn deficiency impacts gene/protein expression.

A crucial protective barrier for the intestinal epithelium is provided by mucus, a viscoelastic hydrogel-like substance that shields the underlying epithelium against chemical, mechanical, and pathogenic damage^4^. Mucus plays an essential role in the absorption of (micro)nutrients, such as Zn. In fact, mucus can bind TEs (reviewed in^5^), prolonging their residence time at the epithelial surface and thereby increasing their bioavailability^6,7^. Intestinal mucus consists of 90–95% water and up to 5% mucins^8^, highly glycosylated proteins classified as either secreted or membrane-bound. To date, 21 mucins belonging to the MUC gene family have been identified^8^, of which MUC2 is the main secreted mucin produced by goblet cells in the small and large intestine^4^. Mucin biosynthesis is a complex, multistep process involving dimerization and extensive *N*- and *O*-glycosylation^9^. *N*-glycosylation initiates in the endoplasmic reticulum (ER), where mannose (Man)-rich glycans are assembled and processed before being transported to the Golgi apparatus for conversion into complex and hybrid type *N*-glycans (Fig. 1A). Additionally, the Golgi apparatus serves as the primary site for *O*-*N*-acetyl-galactosamine (GalNAc) glycosylation, which is initiated by the attachment of GalNAc to Ser/Thr-residues^10^. Driven by the activity and presence of specific glycosyltransferases (GTs), this so-called Tn antigen is extended to up to 8 different core *O*-glycan structures, which can be further elongated and capped by the addition of galactose (Gal), fucose (Fuc) and/or sialic acid (NeuAc) (summarized in Fig. 1B)^8^. In particular, *O*-glycosylation is crucial for mucins’ high water-binding capacity, leading to the hydrogel-like viscoelastic properties of mucus and protecting the protein backbone against bacterial degradation^8^. The produced *O*-glycome plays a vital role in host-microbiome cross-talk, serving as a primary determinant of gut microbial composition and diversity^11^. Accordingly, goblet cells are essential regulators of intestinal homeostasis, playing a crucial role in epithelial protection, immune defense, and nutrient absorption via mucus production. Disruption of mucin production or glycosylation can impair the mucus barrier^12^ and has been linked to the pathogenesis of intestinal disorders, including inflammatory bowel disease (IBD)^12^, and increased susceptibility to infection^13^.

In the 1970 s, changes in mucin composition were reported in Zn-deficient (ZD) rats and sheep, with an increase in mucin sialylation observed under ZD conditions^15^. Previous work from our group showed that, in the HT-29-MTX goblet cell model, the *O*-glycosylation pattern of secreted mucins was profoundly altered during Zn deficiency, with a reduction in more complex *O*-glycan structures^16^. In addition, the mRNA expression of several GTs was deregulated, and *MUC2* was upregulated in ZD cells^16^. Despite these observations, the molecular mechanisms linking Zn deficiency to altered mucin glycosylation and production are still not understood. Furthermore, no information exists on how the *N*-glycan pattern changes during Zn deficiency. This would be highly relevant as *N*-glycosylation is not only important for mucin maturation^17^ but also essential for maintaining cellular homeostasis and health, as it is crucial for determining protein structure and stability, modulating protein expression, and various cellular processes, including cell-cell communication, immune response, and protein folding^10^. The importance of (sub)cellular Zn status for glycosylation was indicated as knocking-out (KO) the Zn transporter solute carrier (SLC)39A9 (ZIP9), which resides in the secretory pathway^18^, led to diminished formation of core 1 *O*-glycans and complex type *N*-glycans^18,19^. Moreover, reduction of Golgi-stored Zn by KO of ZnT7, which shuttles Zn back into the Golgi^20,21^, altered *N*-glycosylation in vitro^22^. However, it remains unclear how a (sub)cellular Zn deficit alters the expression or activity of GTs and how these changes impact mucin structure and composition. Therefore, the aim of this study was to examine the role of Zn in mucin formation and glycosylation at the cellular level using the HT-29-MTX goblet cell model. We investigated how Zn deprivation affects human goblet cells’ Zn homeostasis, mucin production, and global glycosylation patterns by applying a transcriptomic approach to analyze changes in mucins, GTs, and Zn transporters. Additionally, we employed mass spectrometry (MS)-based techniques to screen the changes in mucinome and glycan composition of cellular and secreted mucins.

## Results

### The Zn level in the secretory pathway of goblet cells is diminished in ZD cells

**Fig. 2:**
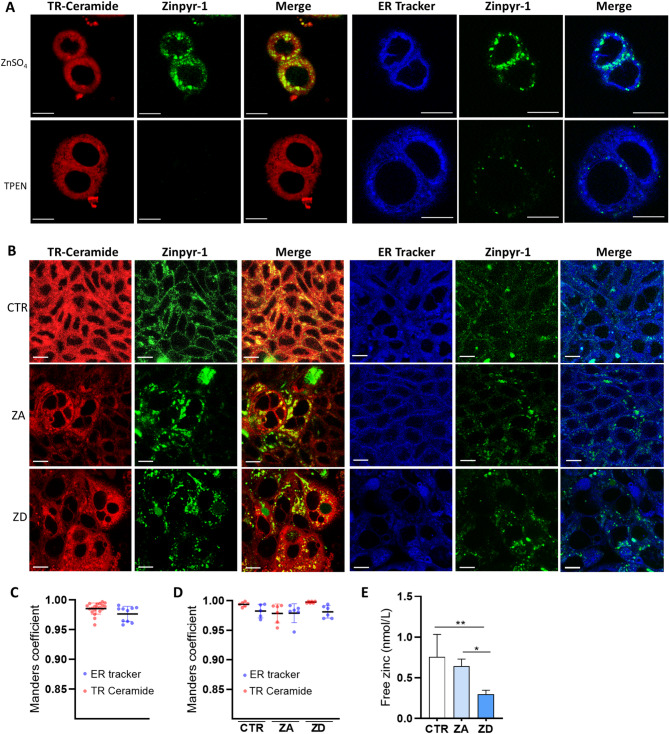
Changes of subcellular free Zn after Zn deprivation and localization of ZP1 in the secretory pathway of the HT-29-MTX goblet cell model. (**A**, **B**) LSM images show the subcellular distribution of free Zn (green) detected with the fluorescent Zn sensor ZP1 and either the Golgi apparatus labelled with the BODIPY™ TR coupled sphingolipid ceramide (red) or the ER stained with ER-Tracker™ Blue-White DPX in CTR HT-29-MTX cells, cultured for 2 d and treated with ZnSO₄ or the Zn chelator TPEN (**A**). Organelle distribution was additionally confirmed in CTR, ZA, and ZD cells upon 14 d cultivation (**B**). Images are representative of *n* = 3–4 independent experiments. Co-localization of the two organelle probes with ZP1 was supported by a Mander’s overlap coefficient (MOC), comparing data of 6–20 images, analyzed in *n* = 3–4 replicates (**C**, **D**). Presented is the quantification of intracellular free Zn in CTR, ZA, and ZD HT-29-MTX cells, measured using the fluorescent Zn probe ZP1 in a 96-well approach (**E**). Data are presented as mean + SD of at least *n* = 3 replicates. Statistically significant differences between treatments were tested with one-way ANOVA followed by Tukey post hoc test (*p < 0.05, **p < 0.01). Scale bar 10 μm.

To assess Zn levels within the secretory pathway, we utilized the low-molecular-weight fluorescent probe Zinpyr-1 (ZP1), which demonstrated significant co-localization with BODIPY™ TR Ceramide and ER tracker in living HT-29-MTX cells (Fig. 2A-B). Co-localization analysis shows proportional co-distribution of both markers with ZP1, as at least 98.5% or 97.6%, respectively, of ZP1 correlates with the stained secretory pathway confirming that the ZP1 signal accurately reports the Zn status of the secretory pathway (Fig. 2A). The specificity of the ZP1 probe for Zn was validated using the cell-permeant chelator Tetrakis-(2-pyridylmethyl)ethylendiamin (TPEN), which successfully abolished the fluorescent signal. Of note, to induce Zn deficiency, cells were treated with Zn-depleted medium using Chelex^®^. To control for changes caused by treating medium with Chelex^®^ resin that were not attributable to Zn-depletion, cells were cultured with Chelex^®^-treated medium with replenished Zn content (Zn-adequate (ZA)). Cellular distribution of ZP1 did not change with cellular Zn-content, as ZP1 correlates with both TR-Ceramide and ER-marker in control (CTR), ZA and ZD cells (TR-Ceramide: 99.4%, ZA 98.3, ZD 99.8%; ER-tracker: CTR 98.2%, ZA 97.7%, ZD 98%, Fig. 2D). In accordance with the significantly reduced total cellular Zn content previously observed in ZD goblet cells^16^, the concentration of free Zn was found to be markedly decreased in ZD HT-29-MTX cells when measured with ZP1 (Fig. 2E). With the spatial analysis of the sensor, this demonstrates that Zn in the secretory pathway was decreased.

### The expression profile of genes essential for cellular Zn homeostasis and mucus synthesis in goblet cells is affected by Zn deprivation

**Fig. 3:**
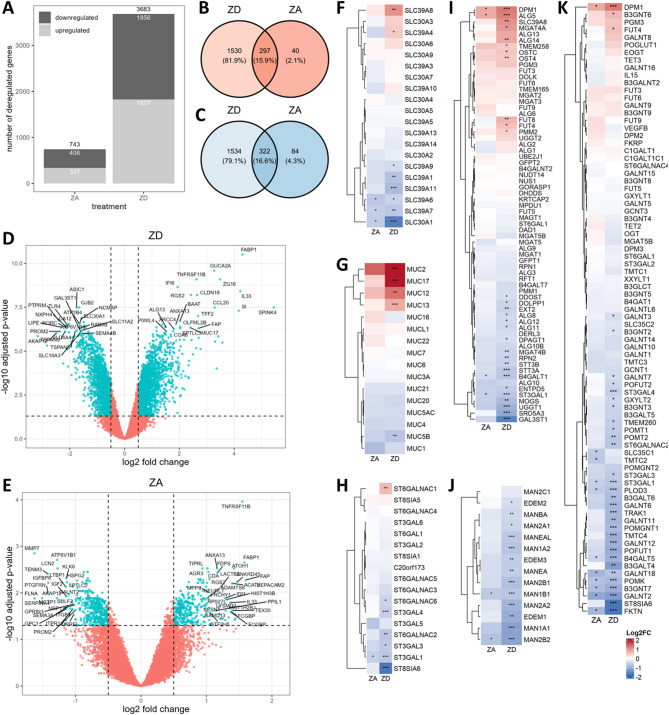
Gene expression profiling of Zn-deficient HT-29-MTX cells. For transcriptomic analysis, HT-29-MTX cells were cultured for 12 days with CTR, ZA and ZD medium. Summary plot quantifying the number of either up- or downregulated genes during ZA and ZD conditions (**A**). Venn diagram presenting the overlap of differentially expressed genes in ZA and ZD cells, upregulated (**B**) or downregulated (**C**). Volcano plots of ZD (**D**) or ZA (**E**) cells illustrate the distribution of significantly up- and downregulated genes based on log₂ fold-change and adjusted *p*-value. Heatmaps shows mRNA deregulation of Zn transporters (GO:0006829; only SLC30 and SLC39) (**F**), mucins (GO: gene-symbols with MUC) (**G**), sialyltransferases (GO:0008373) (**H**) and of proteins important for *N*-glycosylation (GO:0006487) (**I**), mannosidase activity (GO: 0015923) (**J**) and *O*-glycosylation (GO:0006493) (**K**). The expression levels are shown as log₂ fold-change relative to CTR cells; red indicates upregulation, while blue indicates downregulation. Significant differences are indicated (*p_adj_ < 0.05, **p_adj_ < 0.01, ***p_adj_ < 0.001).

To investigate how Zn deprivation affects cellular Zn homeostasis and mucin glycosylation processes in the human goblet cell model HT-29-MTX, the expression profiles of genes involved in Zn transport, mucin production, and glycosylation were analyzed using a transcriptomic approach. This discovery-based approach allowed us to identify candidate genes and pathways for further analysis. In total 3683 genes in HT-29-MTX cells were altered by the ZD conditions, with almost as many genes being up- (1827) as downregulated (1856) compared to CTR cells cultured with standard medium (Fig. 3A). ZA treatment of cells also affected the expression of 743 genes compared to CTR (Fig. 3A). Even though the expression of almost 5-fold more genes was affected by the lack of Zn, than by Chelex^®^-treated medium alone, around 32% of genes were changed in both ZD and ZA conditions, again with an equal proportion of genes being decreased and increased in expression (Fig. 3B-C). The volcano plot allows for visualization of the magnitude and significance of altered gene expression in ZD (Fig. 3D) and ZA (Fig. 3E) cells relative to CTR, underscoring the greater number of alterations in Zn-deprived cells. Accordingly, in ZD cells, the increase in mRNA levels of the *fatty acid binding protein 1* (*FABP1*), important for lipid homeostasis of long chain fatty acids^23^, *guanylate cyclase activator 2 A* (*GUCA2A*), *zymogen granule protein 16* (*ZG16*), *interleukin-33* (*IL-33*) and *Ser peptidase inhibitor kazal type 4* (*SPINK4)*, and *tumor necrosis factor* (*TNF*) *receptor superfamily member 11b* (*TNFRSF11B*) were most pronounced (Fig. 3D). In ZA states, changes in mRNA levels compared to CTR were considerably smaller than in ZD cells, with *TNFRS11B* and *FABP1* being slightly upregulated and *matrix metallopeptidase 7* (*MMP7*), a proteolytic enzyme important for breakdown of extracellular matrix^24^, being the most downregulated (Fig. 3E). Filtering the data set for specific keywords involved in Zn transport or mucin synthesis and glycosylation helped visualize up- and downregulation in ZD and ZA cells (Fig. 3F-K). Among the regulatory proteins for Zn homeostasis, the Zn transporters (GO:0006829) *ZIP8* (*SLC39A8*) and *ZIP4* (*SLC39A4*) were significantly elevated under ZD conditions. In contrast, mRNA levels of *ZnT1* (*SLC30A1*), *ZIP1* (*SLC39A1*), *ZIP6* (*SLC39A6*), *ZIP7* (*SLC39A7*), *ZIP9* (*SLC39A9*), and *ZIP11* (*SLC39A11*) were significantly lower than in CTR conditions (Fig. 3F). ZA treatment showed no upregulation of transporters, only *ZnT1* mRNA was significantly reduced, but to a lesser extent than under ZD conditions.

Concerning mucins (key word: gene symbol MUC), *MUC2*, *MUC12*, *MUC13*, *MUC17*, and *MUC20* mRNAs were significantly increased in ZD cells, while *MUC5B* mRNA was significantly reduced with no significant changes in ZA cells (Fig. 3G). Separate filtering for sialyltransferases (GO:0008373) highlighted the impact that Zn deprivation has for this group of terminating enzymes, as several genes encoding sialyltransferases crucial for terminating both *N*- and *O*-glycans were strongly deregulated in ZD cells (Fig. 3H). Here only *ST6 N-acetylgalactosaminide α−2*,*6-sialyltransferase* (*ST6GALNAC)1* mRNA was significantly elevated, while many others, such as *ST6GALNAC6*,* ST6GALNAC2* and *α-N-acetyl-neuraminide α−2*,*8-*sialyltransferase 6 (*ST8SIA6)* were significantly downregulated. In ZA conditions, *ST3 β-galactoside α−2*,*3-sialyltransferase* (*ST3GAL*)*1*, which catalyzes the transfer of NeuAC to Gal, was the only enzyme whose expression was significantly reduced. Genes involved in the *N*-glycosylation (GO:0006487) exhibited changed gene expression (Fig. 3I). For example, mRNA levels of *fucosyltransferase (FUT)8*, *FUT4*,* and dolichol-phosphate mannosyltransferase subunit 1* (*DPM1*), an important enzyme for formation of mannosyl donors for critical glycosylation pathways, was significantly increased. On the other hand, *mannosyl-oligosaccharide glucosidase* (*MOGS*), which is important for the initiation of *N*-glycan processing^25^, *GAL3ST1*, responsible for sulfation^26^, and *steroid 5 α-reductase 3* (*SRD5A3*), vital for the formation of the oligosaccharide precursor used in the *N*-linked glycosylation^27^, were significantly reduced in ZD conditions. Almost all mannosidases were downregulated on the mRNA level, especially *mannosidase alpha class* (*MAN*) *2B member 2* (*MAN2B2*) and *MAN 1 A member* (*MAN1A*)*1*, crucial for removing Man residues from *N*-glycans in lysosomes or Golgi, respectively^10^ (Figs. 1A and 3J). Genes encoding proteins involved in *O*-glycosylation (GO:0006493) were severely altered, with about 70% of the genes downregulated (Fig. 3K). For example, *ST6GALNAC2*,* protein O-FUT 1* (*POFUT1*), and *β−1*,*4-galactosyltransferase 5* (*B4GALT5*) gene expression was significantly diminished, and genes being significantly upregulated in ZD cells included β−1,3-*N*-acetylglucosaminyltransferase 6 (*B3GNT6)*, *FUT4*, and *DPM1.*

### The mucinome of goblet cells is changed by Zn deficiency

**Fig. 4:**
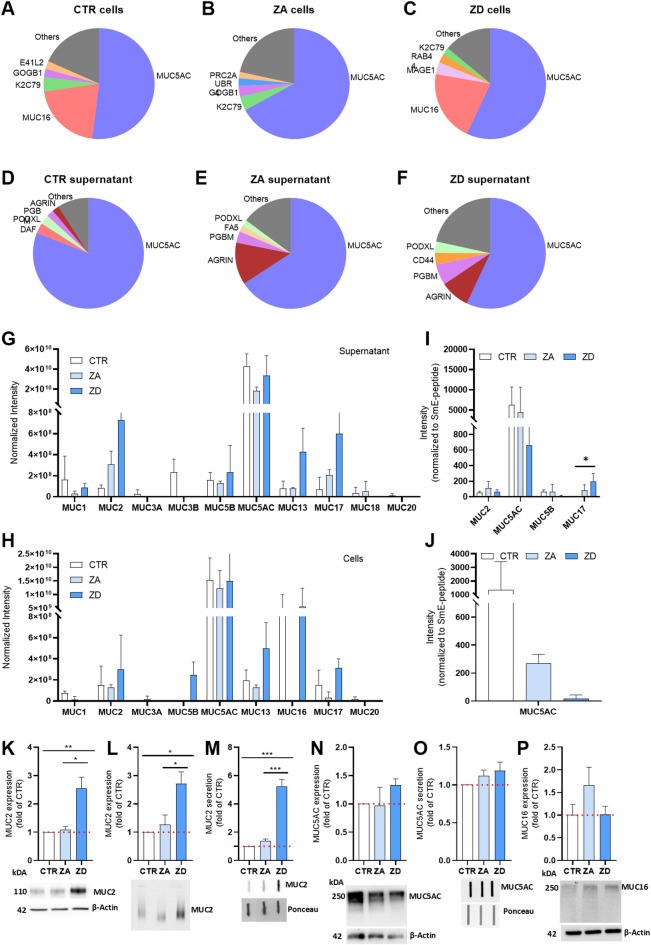
Changes of the mucinome in ZD HT-29-MTX cells. Mucinome of CTR, ZA and ZD HT-29-MTX was analyzed in both cells (**A**–**C**, **H**, **J**) and supernatant (**D**–**I**). Data are presented as mean + SD of at least *n* = 3 biological replicates. Relative distribution of the most pronounced mucin-like proteins is shown in pie charts (A-F). Bar charts depict the normalized expression of canonical mucins in supernatants (**G**) and cells (**H**), including all possible peptides for each treatment, and relative to the SmE-peptide as an internal standard (**I**, **J**). Protein expression of the 110 kDa MUC2-fragment^30^ (**K**), mature MUC2 (**L**), secreted MUC2, MUC5AC (**M**), secreted MUC5AC (**N**), and MUC16 (**O**) are presented. WB data was normalized to the CTR and β-actin, agarose gel and slot-blots to Ponceau staining (representative images of the blots are included). Data are shown as mean (**A**–**F**) and mean + SD (**G**–**N**) of three independent biological replicates. Statistically significant differences between groups were tested with one-way ANOVA followed by Tukey post hoc test (*p < 0.05, **p < 0.01, ***p < 0.001).

The mucinome encompasses all glycoproteins with mucin domains, which are densely *O*-glycosylated and form a bottle-brush-like structure. These include the canonically mucin (MUC) family and other highly *O*-glycosylated mucin-like proteins which are major components of mucus^28^. The mucinome screening of HT-29-MTX cells was carried out using an orthogonal approach that combined enrichment of mucins and mucin-like proteins with a catalytically inactive mutant of the secreted protease of C1 esterase inhibitor (StcE^E447D^) and liquid chromatography-tandem MS (LC-MS/MS). Pie charts allow for visualization of the most abundant mucin-domain glycoproteins detected from enrichment of cells (Fig. 4A-C) and supernatants (Fig. 4D-F). Here, MUC5AC was most pronounced, reaching more than 50% in all three conditions. Otherwise, in the cells, all of the top five most highly expressed mucin-like proteins were different, except for keratin, type II cytoskeletal 79 (K2C79), which was present at comparable amounts (3.2–4.2%). Interestingly, in both CTR and ZD cells (Fig. 4A, C), the second most abundant mucin-like protein was MUC16, which was not detected in ZA cells (Fig. 4B). In the supernatant, AGRIN, podocalyxin-like protein 1 (PODXL), and basement membrane-specific heparan sulfate proteoglycan core protein (PGBM) were expressed in all conditions, where the proportion of these proteins was highest in ZD cells (Fig. 4D-F). Filtering data regarding canonical mucins revealed nine hits among all known mucin proteins in both cells and supernatant, expressed in HT-29-MTX at the mRNA level in Fig. 3G and previous studies^29^. Figures 4G and H show the relative abundance of proteins normalized to all detected canonical mucins. Despite variability among replicates, some trends were observed: for example, MUC2 tended to be increased in ZD supernatants, and both cellular and secreted MUC13 and MUC17 tended to increase under ZD conditions. To allow precise comparison between the different treatment conditions, additional data analysis was performed, focusing only on mucins for which the same peptides were detected across all three treatment conditions and normalized to the same peptide fragment of the mucin-selective metalloproteinase *serratia marcescens* enhancin (SmE) as an internal standard. This reduced the detected MUCs to MUC2, MUC5AC, MUC5B, and MUC17 in the supernatant and MUC5AC in the cell lysates. While the relative increase in MUC2 in ZD supernatant disappeared, the elevation of MUC17 was more pronounced and statistically significant (*p* < 0.05; Fig. 4I) and cellular MUC5AC showed a strong downward trend in ZD cells (Fig. 4J). To additionally confirm changes in MUC2, MUC5AC, and MUC16, we measured protein expression with western blot (WB), agarose gel blot, and slot blot (Fig. 4K-N). Of note, due to the large size of mature MUC2, its 110 kDa C-terminal fragment was measured in WB analysis^30^. Overall, no differences in MUC5AC and MUC16 production were observed across the different conditions, but both the C-terminal fragment and mature MUC2 were significantly overexpressed in ZD cells and also significantly more MUC2 was secreted under ZD conditions, confirming MS results for MUC2, when including all possible MUC2 peptides (Fig. 4H and G), but contradicting those measured for MUC5AC and MUC16.

### Lack of Zn affects the O-glycosylation pattern in goblet cells

**Fig. 5:**
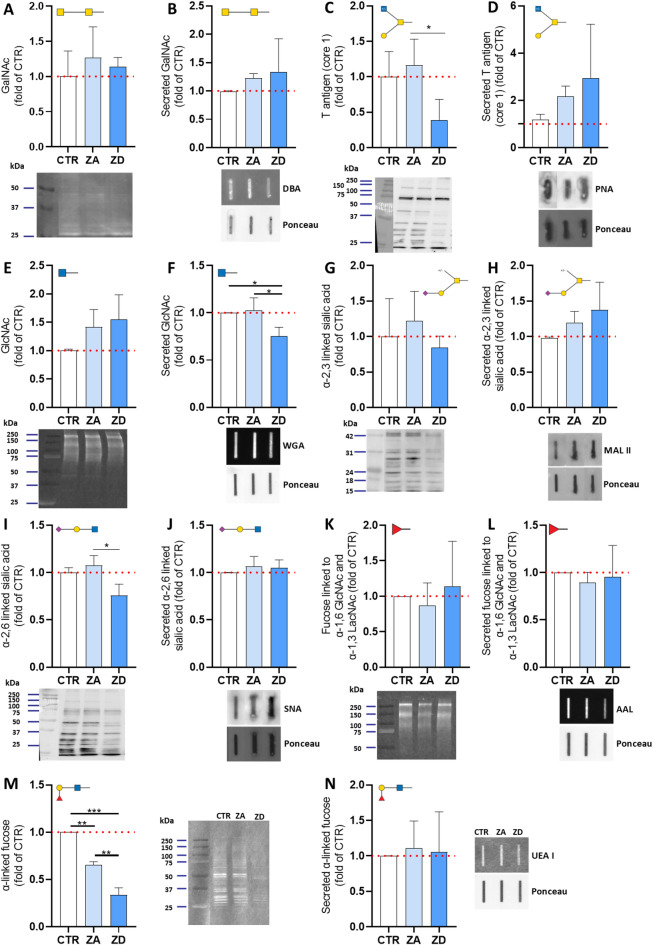
Influence of Zn deficiency on the glycan profile of the HT-29-MTX goblet cell model. Shown are lectin-based measurements of several glycans important for mucin structure found in cells and in secreted mucins of HT-29-MTX cells. The following glycans were analyzed using specific lectins (as listed in brackets): GalNAc (DBA) (**A**, **B**), core-1 (T antigen) (PNA) (**C**, **D**), α-linked GlcNAc (WGA) (E, F), (α- 2,3)-bound NeuAc (MAL II) (**G**, **H**), (α- 2,6) NeuAc (SNA) (**I**, **J**), Fuc linked to α−1,6 GlcNAc and LacNAc (AAL) (**K**, **L**), and α-linked Fuc (UEA I) (**M**, **N**). Data are presented as mean + SD (*n* = 3), analyzed either via lectin blot for cell lysates (A, C, E, G, I, K, M) or slot blot for cell supernatant (B, D, F, H, J, L, N), both normalized to the CTR and the respective ponceau staining (see Supplementary Figure (SFig.) 1). Representative images of the blots are shown on the right side of the corresponding diagram. Significant differences between the treatments CTR, ZA, and ZD are indicated (CTR vs. ZA: **p < 0.01; CTR vs. ZD: *p < 0.05, ***p < 0.001; ZA vs. ZD: *p < 0.05, **p < 0.01; one-way ANOVA with Tukey post hoc test).

Glycans on cellular and secreted proteins were first investigated using lectin blotting, which are glycan binding proteins with specificities towards different glycan structures. Starting with the monosaccharide that initiates protein *O*-glycosylation, we analyzed the levels of GalNAc (also called the Tn antigen) in cells and in the supernatant with *Dolichos Biflorus* agglutinin (DBA) (Fig. 5A, B). Cellular and secreted GalNAc levels were not changed by Zn deprivation. In ZD cells, cellular GalNAc-Gal (core 1), also called T antigen and measured with peanut agglutinin (PNA), (Fig. 5C), was significantly reduced, while the amount of secreted proteins modified with core 1 (Fig. 5D) showed higher variation and was slightly increased after ZD cultivation. *N*-acetylglucosamine (GlcNAc), which is part of core 2–4 *O*-glycans as well as *N*-glycans^31^ and was detected with wheat germ agglutinin (WGA), was not significantly altered but tended to be higher in both ZA and ZD cells (Fig. 5E). On the contrary, in the secreted mucus, the amount of GlcNAc (Fig. 5F) was significantly reduced during Zn deficiency (ZD vs. CTR *p* < 0.05; ZD vs. ZA *p* < 0.05). Terminating oligosaccharides of glycan chains, such as α−2,3- and α−2,6- linked NeuAc, were not changed as measured with *Maackia Amurensis* lectin II (MAL II) or *Sambucus Nigra* lectin (SNA), respectively (Fig. 5G-J). Only cellular *α*−2,6-linked NeuAc was significantly reduced under ZD conditions (Fig. 5I). Moreover, the amount of Fuc linked to α−1,6 GlcNAc and α−1,3 *N*-acetyllactosamine (LacNAc), in cells (Fig. 5K) and supernatant (Fig. 5L), measured with *Aleuria Aurantia* lectin (AAL), was not altered. Particularly, the amount of α-linked Fuc detected with *Ulex Europaeus* agglutinin (UEA I) (Fig. 5M) was significantly decreased in the cells during ZD conditions compared to controls (ZD vs. CTR *p* < 0.001; ZD vs. ZA *p* < 0.01). In addition, ZA-treated cells showed less α-linked fucosylation than CTR, although not as pronounced as in ZD cells (CTR vs. ZA, *p* < 0.01). For the secreted mucus, no alteration was observed in the amount of α-linked Fuc during ZA and ZD treatment (Fig. 5N).

**Fig. 6:**
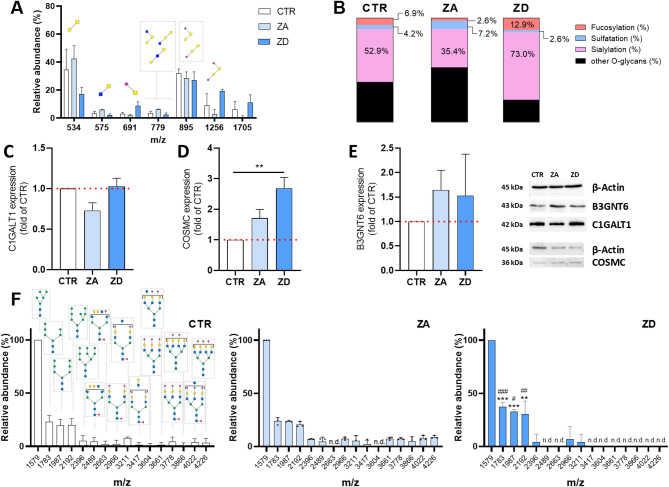
Changes in *O*-glycans of gel-forming mucins and N-glycan profile in ZD HT-29-MTX cells. Shown is the **c**omposition of cellular stored mucin *O*-glycans (**A**, **B**), expression of GTs initiating *O*-glycosylation (**C**–**E**) and *N*-glycans (**F**) in CTR, ZA, and ZD HT-29-MTX cells. Presented are the most abundant *O*-glycans (**A**), the distribution of terminating *O*-glycans (**B**), and cellular *N-*glycans (**F**), analyzed with MALDI-TOF-MS. The protein expression of the GTs C1GALT1 (**C**), the corresponding chaperone COSMC (**D**), and B3GNT6 (**E**) were analyzed via WB normalized to the control and β-actin (representative images of the blots are included). Data are shown as mean (**A**, **B**) and mean + SD (**C**–**F**) of three independent biological replicates. Statistically significant differences were tested with two-way ANOVA (**F**) or one-way ANOVA with Tukey post hoc test (**C**–**E**), respectively, and are indicated ((*p < 0.05, **p < 0.01; in C: ZD vs. CTR with *, ZD vs. ZA with #; # *p* < 0.05; ** or ## *p* < 0.01; *** or ### *p* < 0.01). Predicted *N-*glycan structures are shown above the corresponding m/z values. Glycans were drawn with GlycoGlyph^14^.

The *O*-glycosylation pattern of mucins stored in HT-29-MTX cells was further analyzed by matrix-assisted laser desorption/ionization time of flight (MALDI-TOF)-MS. Figure 6A presents the relative abundance of the *O*-glycan structures, mostly produced in CTR, ZA, and ZD cells. Although the changes were not statistically significant, the relative abundance of unsubstituted core 1, i.e. the T antigen, (534 m/z), decreased in ZD states, while the precursor Tn antigen, in its sialylated form (691 m/z), increased. Moreover, the terminal *O*-glycosylation pattern of cellular mucins was changed by the lack of Zn (Fig. 6B). In particular, sialylation increased in ZD states by approximately 21% (ZD 73.0%, CTR 52.9%), mostly in the amount of sialylated Tn (691 m/z) and disialylated T antigen (1256 m/z; CTR 9.2 ± 14.1%, ZA 2.8 ± 3.3%, ZD 19.3 ± 1.3%), as sialylated T antigen was not changed (895 m/z; CTR 32.0 ± 5.3%, ZA 28.4 ± 4.6%, ZD 27.1 ± 5.9%) (see SFig. 2, Supplementary Table 1). Notably, cultivation of HT-29-MTX cells in ZA medium reduced the total sialylation level to 35.4%. Abundance of fucosylation and sulfation, however, was not changed.

To investigate whether the protein expression of the two GTs glycoprotein-N-acetylgalactosamine 3-β-galactosyltransferase 1 (C1GALT1) and B3GNT6, which are responsible for the formation of core 1 or core 3 *O*-glycans (see Fig. 1B), was also affected by Zn deficiency, similar to what has been observed on the mRNA level (Fig. 3K), we measured their protein expression with WB. C1GALT1 and B3GNT6 (Fig. 6C, E) showed no alterations under ZA and ZD conditions. C1GALT1-specific chaperone 1 (COSMC), essential for C1GALT1 functioning^32^, was significantly upregulated under ZD conditions (*p* < 0.01) (Fig. 6D).

To complement the *O*-glycome data, the *N*-glycome was also assessed using MALDI-TOF-MS. While CTR and ZA HT-29-MTX cells exhibited a broad range of oligomannose and complex branched *N*-glycans (Fig. 6F), ZD cells showed a marked shift toward oligomannose *N*-glycans. In ZD samples, complex *N*-glycans were nearly absent, replaced by dominant oligomannose peaks Man5GlcNAc2 (m/z 1579), Man6GlcNAc2 (m/z 1783), Man7GlcNAc2 (m/z 1988), Man8GlcNAc2 (m/z 2192), and Man9GlcNAc2 (m/z 2396). Notably, the relative abundance of Man6GlcNAc2 through Man8GlcNAc2 was significantly higher in ZD cells than in CTR or ZA controls.

## Discussion

### Mucus composition during zinc deficiency

During Zn deficiency, a decrease in mucus thickness has been described in vivo^15^ and in vitro^16^, resulting in impaired epithelial protection. It has to be noted, that all observations were made with a cancer cell line that predominantly secretes gastric mucins (MUC5AC)^33^. Yet, there are only a few non-malignant human goblet cells available to study cellular mechanisms in vitro and HT-29-MTX cells have been widely used as a model for mature intestinal goblet cells under specific culture conditions^34^ providing a well-characterized model for ZD goblet cells^35^. Our in vitro study allowed us to dissect the observation of decreased mucus during Zn deprivation in more detail and identified changes in the levels of both secreted, gel-forming (MUC2, MUC5AC) and transmembrane (MUC13, MUC17) mucins after Zn deprivation. MUC2 was upregulated not only at the mRNA^16^ but also at the protein level, leading to elevated secretion of the mature protein by ZD cells. And also, the mucinome analysis shows a trend to higher MUC2. One reason why the mucinome analysis did not reveal a significant increase in MUC2 and showed particularly large variation in cells, could be the StcE^E447D^-beads used to isolate mucin-like proteins. The mucinase StcE was found to recognize and bind *O*-glycans with elaborate core 1 and core 2 more efficiently than core 3 *O*-glycans^36^. As the glycosylation pattern of mucins was changed in ZD cells, weaker binding and isolation of these proteins by StcE^E447D^-loaded beads and impaired digestion by SmE could explain the high variance and the diminished amount of MUC2 detected in the samples. Accordingly, impaired isolation due to changes in the glycosylation pattern could also explain the diminished MUC5AC in ZD cells and the complete loss of MUC16 signal in ZA cell samples, as measured by MS but not confirmed by WB or slot-blot analysis.

Additionally, Zn deficiency exerted an inductive effect on MUC13 mRNA and MUC17 mRNA and protein levels. Both transmembrane mucins play an integral role in intestinal immune defense and epithelial maintenance during inflammation^37,38^. Heightened *MUC13* production in ZD cells could be linked to the elevated membrane permeability observed during Zn deficiency^39^, as MUC13 was recently shown to modulate barrier integrity by inversely regulating the expression of tight junction proteins^38^. In principle, although the mucus layer decreases, transcriptome, WB, agarose gel, slot-blot analyses, and in part the mucinome analysis, strongly indicate that the expression of certain mucins (MUC2, MUC17) was induced in ZD cells, potentially to compensate for the altered *N*- and *O*-glycosylation of these glycoproteins. Overall changes in mucin production caused by Zn deprivation could be explained by the (pro)inflammatory milieu, as Zn deficiency is characterized by increased production of reactive oxygen species^40^ and (pro)inflammatory cytokines^41^, which has been shown to induce mucin expression, such as MUC17 and MUC5AC^37,42^. Still, further research is needed to clarify these changes in intestinal goblet cells and to understand the role of Zn in mucin synthesis.

### Cellular zinc homeostasis during zinc deficiency

Prolonged Zn deprivation of HT-29-MTX altered their cellular Zn homeostasis, which was reflected in a pronounced decrease of total^16^ and subcellular free Zn concentration. Zn transporters in the intestine are regulated in response to low available Zn concentrations, as described in vitro^43,44^, and in vivo^45–47^, reflecting a counteracting mechanism against low Zn status to maintain body Zn homeostasis^48^. While this has been thoroughly studied in the intestine and absorptive intestinal cells (reviewed in^3^), there is still limited information on the cellular Zn homeostasis in non-enterocyte cell types of the intestinal epithelium. Thus, screening the goblet cell in vitro model system HT-29-MTX provides insight into potential changes in this secretory cell under ZD conditions. The primary Zn transporters in the intestinal tract, ZIP4 and ZnT1, which control both cellular Zn levels and overall systemic Zn balance, were deregulated. Additionally, expression of transporters of the secretory pathway was altered, with both *ZIP7*, the only Zn transporter identified so far to sequester Zn from the ER^49^, and *ZIP9*, secreting Zn from the Golgi back into the cytoplasm^50^, being significantly downregulated. Changes in Zn concentrations within the secretory pathway have been shown to affect proper glycosylation^18,19,22^. In particular, ZIP9 was identified as a regulator of both *N*- and *O*-glycans, as ZIP9 KO cells exhibited a perturbed *N*-/*O*-glycan pattern^18,19^. Thus, the decrease in *ZIP7* and *ZIP9* mRNA might indicate a counteracting mechanism to restore ER and Golgi Zn levels and maintain their function. Accordingly, subcellularly stored free Zn, measured with the fluorescent Zn-sensor ZP1, was drastically reduced in ZD cells. In fact, ZP1 seemed to accumulate in the secretory pathway, confirming perturbed Zn homeostasis in the Golgi and ER.

### O- and N-glycosylation of mucins and smaller glycoproteins during zinc deficiency

The cellular *O*-glycosylation pattern changed during ZD cultivation, even though results were not significantly different. In supernatants of HT-29-MTX much lower core 1 and higher abundance of unsubstituted core 3 (575 m/z) were reported^16^. In cell lysates, however, we mostly detected a tendency of decrease in unsubstituted core 1 in ZD states. Transcriptome analysis of various GTs involved in *N*- and *O*-glycosylation showed notable changes under Zn deficiency. As core 1 and 3 *O*-glycans share the same precursor, the Tn antigen, the overall reduction in T antigen observed in ZD cells, not only in mucins but also in smaller glycoproteins, could be explained by competition between specific GTs^51^. Accordingly, the transferase B3GNT6, responsible for forming core 3 structures, was elevated at the transcript level in ZD conditions, confirming previous *q*PCR studies^16^. However, no differences were found at the protein level. Core 1 *O*-glycans are catalyzed by C1GALT1 and its chaperone COSMC^52^, which is required for C1GALT1 functioning and essential for the synthesis of about 80% of Muc2 *O*-glycans^32^. While no changes of *C1GALT1* and *COSMC* were detected in the transcriptomic screening, COSMC was significantly increased in ZD cells. This seems unexpected, as their main product, the T antigen, decreased in ZD cells. A possible explanation could be that COSMC, a Zn-binding protein, does not function properly without Zn, as Zn-binding stabilizes its native form and may affect its activity^52^. Hanes et al. observed that metal chelation destabilized the oligomeric form of COSMC (33 kDa), suggesting that both oligomerization and metal binding might regulate its function^52^. Given that Zn levels in the secretory pathway were decreased in ZD HT-29-MTX cells and free Zn is crucial for ER proteostasis and functioning^53,54^, depleted free Zn in these organelles might lead to impaired proper oligomerization of COSMC, leading to upregulation on the protein level to compensate for its reduced function.

In addition to the increase in truncated glycans, particularly present in mucins secreted by ZD goblet cells^16^, the composition of terminal glycans seemed to be altered. This supports the idea that Zn deficiency changes the expression of GTs or/and that a lack of Zn in the secretory pathway might also impair the functioning of GTs^55^. Zn deprivation led to a higher abundance of sialylated mucin *O*-glycans, particularly sialylated Tn and disialylated core 1 (T antigen). This was consistent with observations in COSMC KO mice^32^, which have been characterized by elevated levels of Tn and sialylated Tn^32^, again supporting the theory that COSMC is not functioning properly in ZD cells. Furthermore, the increase in sialyl-Tn was also reflected in significantly elevated *ST6GALNAC1*, which catalyzes the transfer of NeuAc, in an −2,6 linkage to *O*-linked GalNAc/Tn antigen^56^, while all other sialyltransferases decreased on the mRNA level. Evidence suggests that sialylation of GTs also affects their (sub)cellular localization and function^56^, which further impacts glycosylation processes.

Glycosylation of mucins, especially terminal glycans, e.g. Fuc and NeuAc, and T antigen formation, has been described as essential for intestinal homeostasis^56,57^. Additionally, alterations in *O*-glycan composition could impair uptake of trace elements, as observed during Zn deficiency^58^, as the high metal-binding capacity of mucus is believed to primarily involve the carboxyl and *N*-acetyl groups of glycans^7^. Further, the formation of a gel-like protective network and interactions with microbiota can be affected by altered *O*-glycan composition, resulting in a thinner, less protective mucus layer^59^, potentially increasing the pathogen burden. Thus, to the extent that the observations made in vitro can be translated to the in vivo situation, all of this could be substantial for intestinal health and could ultimately explain the heightened susceptibility to disease in people with moderate Zn deficiency, and likewise pertains to the substantial increase in diarrhea cases associated with prolonged severe Zn deficiency^2^.

Lectin blot analysis did not reflect the increase in sialylation measured by MALDI-TOF. Also, in supernatants analyzed by lectin slot blots, no decrease in sialylation was observed, in contrast to the instrumental analysis of secreted mucins from ZD HT-29-MTX^16^. It should be noted that WB-based lectin blots are limited in their ability to detect large proteins, thereby restricting the analysis of glycoproteins larger than ∽250 kDa (e.g., mucins). However, this may account for the differences observed at the cellular level, but not those detected in the supernatant measured via slot blot. Unlike MALDI-TOF analysis, lectin detection of specific glycans has been shown to depend on the surrounding moieties of the respective glycans^60^. For example, MALII recognizes (α-2,3) bound NeuAc but also sulfated Gal-residues and is inhibited by Fuc in underlying residues^60^, making it difficult to only rely on lectin-assays for complex glycan structures like mucins. Further, PNA was found to only detect the T antigen when the Gal-residue is unsubstituted^60,61^, and not sialylated, making the results obtained with this lectin difficult to interpret. However, lectin-WB glycan analysis clearly shows that, in addition to mucins, smaller, possibly less complex glycosylated glycoproteins are affected by Zn deficiency, particularly in their Fuc and T antigen content. In addition to GTs, glycosidases^10^, such as fucosidases, and Zn-dependent peptidases (such as matrix metalloproteinases (MMPs)^62^), could also be affected by Zn deficiency, altering the final glycan pattern and structure of mucins or glycoproteins in general. Future research should therefore also include the impact of Zn on those enzyme classes, identify these smaller glycoproteins and investigate their sugar coating with instrumental techniques. One possibility is that these smaller bands correspond to *N*-glycosylated proteins, as they are typically smaller in size and *N*-glycan formation was similarly affected in ZD cells. CTR and ZA goblet cells produced complex *N*-glycans, corresponding to fucosylated and sialylated moieties, which were formed in the *trans* Golgi^10^. In contrast, only mannosylated *N*-glycan forms produced in the ER and *cis* Golgi were present upon Zn deprivation, with products originating from the *medial* Golgi absent and only very low levels of two complex *N*-glycans formed in the *trans* Golgi^10^. During Zn deficiency, mRNA levels of the mannosidases responsible for trimming the glycoprotein during entry into the Golgi (*Man1A1*, *Man1A2*), as well as GnT-IV (*MGAT4*), were significantly reduced, possibly explaining the decrease in more complex *N*-glycan structures in ZD cells. Furthermore, human MAN2A1 is known to require Zn for its activity^22^, as further evidenced by the presence of Zn in the active site of its Drosophila homologue^63,64^. Accordingly, a lack of available Zn in the Golgi could directly disrupt the formation of hybrid and complex type *N*-glycans. This, of course, could have significant consequences for various physiological processes^10^. Regarding mucins, aberrant *N*-glycosylation could impair their maturation, folding, secretion, or membrane retention, thereby compromising the barrier function^12,65^, in addition to observed MUC expression changes. Thus, a thorough investigation of the structural and functional changes of both transmembrane and secreted mucins caused by Zn deficiency should follow.

## Conclusion

Taken together, Zn deficiency altered the composition of mucus, including the quantity and types of mucins present, their *N*-glycosylation as well as tendencies toward altered cellular *O*-glycosylation, supporting the idea that intestinal symptoms associated with Zn deficiency are also attributable to impaired mucus production by goblet cells. Ultimately, this study emphasizes the critical role of Zn in *N*- and *O*-glycosylation, positioning it alongside manganese and calcium as a key metallic regulator of the glycome^66^.

## Materials and methods

### Materials

Fluorescein isothiocyanate (FITC)-labelled AAL (FL-1391-1, Vector Laboratories, Inc., Newark, NJ, USA), anti-B3GNT6 (21291-1-AP, Proteintech, Rosemont, IL, USA), bicinchoninic acid (BCA) (Sigma Aldrich, St. Louis, MO, USA), anti-biotin HRP-linked antibody (#7075, Cell Signaling Technology, Danvers, MA, USA), BlueElf Prestained Protein Marker (Jena Bioscience, Jena, DE), bovine serum albumin (BSA) (Sigma Aldrich, St. Louis, MO, USA), anti-C1GALT1 (sc-100745, Santa Cruz Biotechnology, Dallas, TX, USA), anti-COSMC (sc-271829, Santa Cruz Biotechnology, Dallas, TX, USA), BODIPY™ TR Ceramide (D7540, Invitrogen™, Carlsbad, CA, USA), Chelex^®^ 100 Resin (Bio-Rad, Hercules, CA, USA), Tetramethylrhodamine isothiocyanate (TRITC)-labelled DBA (21761016-1, Glycomatrix™, Dublin, OH, USA), Dulbecco’s Modified Eagles Medium (DMEM) (PAN-Biotech, Aidenbach, DE), Fetal calf serum (FCS) (Serana Europe GmbH, Pessin, DE), Fluoromount G™ (Invitrogen™, Carlsbad, CA, USA), Hoechst 33,258 (Sigma Aldrich, St. Louis, MO, USA), biotinylated MAL II (B-1265, Vector Laboratories, Inc., Newark, NJ, USA), Vivaspin^®^ Turbo 15 100 kDa molecular weight cut off (MWCO) filter (Sartorius, Göttingen, DE), anti-mouse IgG HRP-linked antibody (#7076, Cell Signaling Technology, Danvers, MA, USA), anti-MUC2 (ab134119, Abcam, Cambridge, UK), anti-MUC5AC (MA5-12178, Thermo Fisher Scientific, Waltham, MA, USA), anti-MUC16 (MABC1608, Sigma Aldrich, St. Louis, MO, USA), non-essential amino acids (NEAA) (Sigma Aldrich, St. Louis, MO, USA), 0.45 μm nitrocellulose (NC) membrane (Cytiva Europe GmbH, Munich, DE), biotinylated PNA (B-1075, Vector Laboratories, Inc., Newark, NJ, USA), penicillin and streptomycin (Sigma Aldrich, St. Louis, MO, USA), 0.2 μm polyvinylidene difluoride (PVDF) membrane (GE Healthcare Life Science, Chicago, IL, USA), Ponceau S (Carl Roth GmbH & Co. KG, Karlsruhe, DE), anti-rabbit-Alexa Fluor^®^ 488 (preadsorbed) (ab150077, Abcam, Cambridge, UK), anti-rabbit IgG HRP-linked antibody (#7074, Cell Signaling Technology, Danvers, MA, USA), biotinylated SNA (B-1305, Vector Laboratories, Inc., Newark, NJ, USA), Skimmed milk powder (Carl Roth GmbH & Co. KG, Karlsruhe, DE), SuperSignal™ West Pico PLUS (Thermo Fisher Scientific, Waltham, MA, USA), Triton X-100 (Sigma Aldrich, St. Louis, MO, USA), FITC-labeled UEA I (FL-1061, Vector Laboratories, Inc.; Newark, NJ, USA), FITC-labeled WGA (L4895, Sigma Aldrich, St. Louis, MO, USA), ZnSO_4_ × 7H_2_O (Sigma Aldrich, St. Louis, MO, USA). All other chemicals were purchased from standard sources.

### Cell culture

The human colon adenocarcinoma cell line HT-29-MTX-E12 was obtained from the European Collection of Authenticated Cell Cultures (ECACC, Porton Down, UK; ECACC 12040401). Cells were cultured in DMEM with phenol red and 4.5 g/L glucose, supplemented with 10% FCS and 100 U/mL penicillin and 100 µg/mL streptomycin, and 1% NEAA (CTR medium) at 37 °C with 5% CO_2_. ZA and ZD media were generated with Chelex^®^ 100 Resin as described^16^-Briefly, CTR medium was depleted of divalent trace metals with Chelex^®^ 100 Resin (50 g/L medium) (Zn content of CTR medium: 126 ± 19.3 µg/L; ZD medium: 7.5 ± 2.8 µg/L (see SFig.3) via inductively coupled tandem MS (ICP-MS) (8800 ICP-QQQ-MS, Agilent Technologies, Waldbronn, DE)). Essential TE, which were also removed by Chelex^®^-treatment (see SFig.3), were replenished to complete medium conditions. Chelex^®^ medium was sterile-filtered (0.2 μm cut-off filter, Sigma Aldrich, St. Louis, MO, USA). ZA medium was prepared by adding the amount of removed Zn, back into ZD medium.

For lectin blot, slot blot, agarose gel, sodium dodecyl sulfate (SDS)–polyacrylamide gel electrophoresis (PAGE), as well as mucinome and MALDI-TOF analysis, cells were cultured in 150 cm² dishes (initial cell number: 2 × 10^6^ cells/dish) for 14 days. For free Zn analysis, cells were cultured in a 96-well plate (initial cell number 5 × 10^4^ cells/well). One day after seeding, cells were incubated with CTR, ZA, and ZD media, respectively, the media were changed every 2–3 days. Twenty-four hours before collecting samples, media were changed to DMEM w/o phenol red (100 U/mL penicillin, 100 µg/mL streptomycin, 0% FCS). Secreted mucus was harvested by gently washing the cell layer with the supernatant to detach secreted mucus from the cells. Supernatant with mucus was concentrated 4-fold using 100 kDa MWCO filters. Cells were scraped using 750 µL of phosphate-buffered saline (PBS). Samples were stored at −80 °C until further analysis.

### Transcriptome analysis

Cells were cultured with the respective media CTR, ZA, ZD for 13 days. Media was discarded, cells were washed with ice-cold PBS, scrapped and collected in ice-cold PBS, spun down at 0.8 *g* 4 °C and pellets were stored at −80 °C and shipped. The samples were processed and labeled with a GeneChip WT Plus labeling kit at Eurofins (Aarhus, Dk). The microarray analysis on Human Clariom S Assay HT assays (Thermo Fisher Scientific, Waltham, MA, USA) was performed at Eurofins Genomics Europe Genotyping A/S (Eurofins, Aarhus, Dk). The raw data of this publication have been deposited in NCBI’s Gene Expression Omnibus and are accessible through GEO Series accession number GSE319402.

Statistical data analysis and visualization was performed using R, version 4.3.2^67^. The raw data (CEL files) were normalized and summarized using the RMA (robust multiarray average) algorithm from the R package oligo version 1.66.0. The genes were associated with vendor-provided annotation information [Release 36 (Thermo Fisher)] and control genes were removed after background correction. Moreover, irrelevant genes with low or unchanged expression were removed so that 17,286 probes corresponding to 15,997 genes remained (intensity > 3 in at least 2 samples). Differential gene expression analysis was performed by the R package limma version 3.58.1^68^. Linear models were fit and moderated t-statistics were computed by the eBayes function. Afterwards, the Benjamini-Hochberg correction was applied to exclude false-positive results, and thus differentially expressed genes with an adjusted p-value < 0.05 and absolute log_2_ fold-change > 0.5 were considered significant. Probabilistic Principal Component Analysis (PCA) was applied on pareto-scaled and scaled data by the R package pcaMethods version 1.94.0^69^. Heatmaps were generated by the R package ComplexHeatmap version 2.18.0^70^ using default settings if not mentioned otherwise. Intersections of gene sets were visualized as UpSet plots using R package UpSetR version 1.4.0. Comparison analysis to predict diseases and functions were performed and the resulting z-score was visualized as heatmaps. Gene ontology (GO) Biological Process term enrichment analysis was performed using a hypergeometric test by the R package clusterProfiler version 4.10.0^71^, and false discovery rate (FDR) was applied to control for multiple testing.

### Cellular free Zn analysis

Free Zn was quantified with the Zn-specific fluorescent probe ZP1 (Santa Cruz biotechnology, Dallas, TX, USA) as described^6^. Briefly, CTR, ZA, and ZD cells were incubated with 2.5 µM in free Zn assay buffer (NaCl 120 mM, KCl 5.4 mM, glucose 5 mM, CaCl_2_ 1 mM, MgCl_2_ 1 mM, NaH_2_PO_4_ 1 mM, 4-(2-hydroxyethyl)−1-piperazineethanesulfonic acid (HEPES) 10 mM, pH = 7.35) with 3 g/L BSA for 30 min at 37 °C. In order, to quantify free Zn concentration, the equation of Grynkiewicz et al.^72^. was applied using the minimal fluorescence (Fmin) of the sensor, induced with 20 µM of the Zn chelator TPEN, and the maximal fluorescence (Fmax) by incubating the cells with 600 µM ZnSO_4_. Fluorescence was measured at λ_Ex_ = 508 nm, λ_Em_ = 527 nm using a microplatereader (Spark, Tecan Group, Männedorf, Switzerland).

### Confocal laser scanning microscopy

Confocal laser scanning microscopy (LSM) was performed using HT-29-MTX cells seeded at a density of 2.5 × 10⁴ cells per well in ibidi µ-Dishes (#80806, Ibidi GmbH, Gräfelfing, DE) and cultured for 48 h. On day two, cells were incubated with 2.5 µM ZP1 (λ_Ex_ = 488 nm, λ_Em_ = 490–579 nm) in the above-described free Zn assay buffer with 3 g/L BSA for 30 min at 37 °C to visualize intracellular free Zn. After washing the cells with assay buffer, cells were incubated for additional 30 min at 37 °C with 5 µM BODIPY-labelled sphingolipid BODIPY™ TR Ceramide (λ_Ex_ = 594 nm, λ_Em_ = 599–734 n; #D7540, Invitrogen™, Carlsbad, CA, USA) or 500 nM ER-Tracker™ Blue-White DPX (λ_Ex_ = 405 nm, λ_Em_ = 410–480 nm; #E12353 Invitrogen™, Carlsbad, CA, USA) (in Hanksʼ buffered salt solution/HEPES (NaCl 138 mM, KCl 5.33 mM, glucose 5.55 mM, CaCl_2_ 1.26 mM, MgCl_2_ 0.49 mM, MgSO_4_ 0.41 mM, KH_2_PO_4_ 0.44 mM, NaH_2_PO_4_ 0.34 mM, NaHCO_3_ 4.17 mM, HEPES 10 mM, pH = 7.4) to label the Golgi apparatus or ER, respectively. Cells were subsequently washed twice with assay buffer. To validate the sensitivity of ZP1 to Zn, intracellular Zn levels were depleted with 20 µM TPEN. Live cell imaging was performed using a Zeiss LSM 880 (Carl Zeiss Microscopy, Oberkochen, DE) equipped with a Plan-Apochromat 63x/1.4 Oil DIC M27 objective and Argon multiline laser 488 nm, HeNe laser 594 nm and DPSS 561–10 nm laser (Fluorescence channel bleed-through was controlled, see SFig. 4). ZEN software (black edition) system 2.3 SP1 (Zeiss) was used for settings. Image analysis was performed using the open-source program ImageJ/Fiji (version 2.16.0/1.54p, NIH, Bethesda, MD, USA)^73^, and co-localization was analyzed using the JACoP plug-in^74^, calculating the Mander’s overlap coefficient (MOC) for ZP1 overlapping Golgi or ER staining, respectively.

### Electrophoresis and immune blotting

Cells were lysed using radioimmunoprecipitation assay buffer (RIPA) lysis buffer (Tris-HCl 50 mM, NaCl 150 mM, EGTA 2 µM, SDS 0,1%, Triton X-100 1%, deoxycholic acid sodium salt 0,5%) and protein concentration was analyzed by BCA assay as described^75^. For SDS-PAGE 10 µg protein per sample was loaded on 8% or 12% polyacrylamide gels and transferred to PVDF membrane. For agarose gel electrophoresis 50 µg protein per sample was loaded on 0,7% agarose gels. Gel was run at 65 V for 2.5 h and proteins were transferred using capillary force to a 0.45 μm NC membrane as described^76^. Membranes were blocked with either skimmed milk or BSA in Tris-buffered saline with Tween^®^ 20 0,1% (TBS-T). Primary antibodies (MUC2, 1:2000, 5% milk; MUC5AC, 1:1000, 5% BSA; MUC16, 1:1000, 5% BSA) were incubated overnight at 4 °C, and secondary antibodies (anti-rabbit HRP-linked, 1:1000, 5% BSA; anti-mouse HRP-linked, 1:1000, 5% BSA) were incubated for 1 h at RT. Blots were visualized using SuperSignal™ West Pico PLUS reagent. Images were captured using the ChemiDoc™ MP Imager (Bio-Rad, Hercules, CA, USA) and analyzed using ImageJ/Fiji (version 2.16.0/1.54p, NIH, Bethesda, MD, USA).

### Lectin blot

Cell lysis, dilution, and SDS-PAGE procedure was performed in the same way as WB analysis. After gel electrophoresis proteins were transferred to NC membranes and blocked using 3% BSA in TBS-T. Lectins (3 µg/mL: AAL, MAL II, PNA, SNA, WGA; 10 µg/mL: UEA; 30 µg/mL: DBA) were incubated for 30 min in 3% BSA in TBS-T at RT. Biotinylated lectins were subsequently incubated with anti-biotin (1:1000, 3% BSA in TBS-T) for 1 h at RT. Blots with biotinylated lectins were visualized using SuperSignal™ West Pico PLUS reagent. To control for endogenous biotin in the cells, bands with biotinylated proteins were excluded from the data analysis (see SFig. 1H). Endogenous biotin in cell supernatants did not differ between treatments (SFig. 1I).

### Slot blot

Protein concentration from the collected cell supernatant was determined via BCA-assay. Samples were diluted to 2 mg/mL protein and 20 µL were blotted onto a NC membrane. Subsequently, the membranes were stained with Ponceau S and images were acquired. Incubation of lectins and antibodies was performed as described above.

### O-glycan analysis with MALDI-TOF-MS

Cells were purified, and *O*-glycans were released by ß-elimination with a treatment of alkaline borohydride, as previously described^77^. Mucin glycans were permethylated and subsequently analyzed using MALDI-TOF-MS in the ion positive mode^77^ (MALDI-TOF/TOF ABI 4800 ABsciex, Toronto, CA).

### N-glycan analysis

Cells were lysed (1% Triton X-100), sonicated, and proteins were reduced with 10 mM DTT and alkylated with 50 mM iodoacetamide. Proteins were then precipitated with 10% TCA, washed with acetone, and digested with trypsin as previously described^78^. *N*-glycans were enzymatically released using PNGase F and purified by C18 Sep-Pak chromatography. Following lyophilization, glycans were permethylated and analyzed via MALDI-TOF-MS (MALDI-TOF/TOF ABI 4800 ABsciex, Toronto, CA), with spectra accumulated over 10,000 scans across a range of m/z 1500–4500.

### Mucinome analysis with LC-MS/MS

#### Preparation of StcE^E447D^ beads and mucin enrichment

The pET28 plasmids for His-tagged SmEnhancin and StcE were kindly provided by the Bertozzi laboratory. Following the protocol published in Mahoney et al.^79^. StcE^E447D^ was conjugated to NHS-activated beads and washed 3× with 1 mL of PBS followed by the addition of the proteins, which were allowed to bind overnight at 4 °C. Free NHS-esters were capped by adding 100 mM Tris, pH 7.4, to the bead slurry and incubated for 3 h at 4 °C. After capping, beads were washed 3× with a high-salt buffer (20 mM Tris and 500 mM NaCl) followed by a buffer without salt (20 mM Tris). HT-29-MTX cell lysates and media (supernatant) were filtered through a 0.22 μm vacuum filter (Merck Millipore, Billerica, MA, USA), concentrated using Amicon Ultra centrifugal filters (30 kDa MWCO, Merck Millipore, Billerica, MA, USA), and analyzed by BCA assay (Thermo Fisher Scientific, Waltham, MA, USA) to determine protein concentration. 2 mg of samples were brought to a final volume of 500 µL in no-salt buffer, and ethylenediaminetetraacetic acid (EDTA) was added to a final concentration of 1 mM. The protein-conjugated solid supports (100 µL) were added to the samples and allowed to rotate at 4 °C overnight. After binding, the beads were washed 3× with the high-salt buffer and 2× with the no-salt buffer. Bound proteins were eluted by adding 100 µL of 0.5% sodium deoxycholate (SDC) in 20 mM Tris and boiling the beads at 95 °C with agitation. The elution step was repeated once, and the two eluates were pooled.

#### Mass spectrometry sample preparation

Following elution, the enriched samples were subjected to reduction, alkylation, and proteolytic digestion. To begin, dithiothreitol (DTT) was added to a concentration of 1 mM and allowed to react at 65 °C for 20 min followed by alkylation in 1.5 mM iodoacetamide (IAA) for 15 min in the dark at RT. Subsequently, 800 ng of mucinase SmE was added and allowed to react overnight at 37 °C, then subjected to an additional digestion with 100 ng of trypsin (Promega, Fitchburg, WI, USA) for 6 h at 37 °C. All reactions were quenched by adding 1 µL of formic acid (85178, Thermo Fisher Scientific, Waltham, MA, USA) and diluted to a volume of 200 µL prior to desalting. The addition of formic acid also caused SDC to precipitate out of the solution, and the resulting supernatant was transferred to a new tube before desalting. Desalting was performed using 10 mg Strata-X 33 μm polymeric reversed-phase solid-phase extraction (SPE) columns (8B-S100-AAK, Phenomenex, Torrance, CA, USA). Each column was activated using 500 µL of acetonitrile (ACN) (LC015, Honeywell, Charlotte, NC, USA) followed by 500 µL of 0.1% formic acid, 500 µL of 0.1% formic acid in 40% ACN, and equilibration with two additions of 500 µL of 0.1% formic acid. After equilibration, the samples were added to the column and rinsed twice with 200 µL of 0.1% formic acid. The columns were transferred to a 1.5 mL tube for elution by two additions of 150 µL of 0.1% formic acid in 40% ACN. The eluate was then dried by using a vacuum concentrator (LabConco, Kansas City, MO, USA) prior to reconstitution in 8 µL of 0.1% formic acid.

#### Mass spectrometry data acquisition

Samples were analyzed by online nanoflow LC-MS/MS using an Orbitrap Eclipse Tribrid mass spectrometer (Thermo Fisher Scientific, Waltham, MA, USA) coupled to a Dionex UltiMate 3000 HPLC (Thermo Fisher Scientific, Waltham, MA, USA). For each analysis, 2 µL was injected onto an Acclaim PepMap 100 column packed with 2 cm of 5 μm C18 material (164564, Thermo Fisher Scientific, Waltham, MA, USA) using 0.1% formic acid in water (solvent A). Peptides were then separated on a 15 cm PepMap RSLC EASY-Spray C18 column packed with 2 μm C18 material (ES904, Thermo Fisher Scientific, Waltham, MA, USA) using a gradient from 0 to 35% solvent B (0.1% formic acid with 80% ACN) in 60 min. Full scan MS1 spectra were collected at a resolution of 60,000, an automatic gain control target of 3e5, and a mass range from *m/z* 300 to 1500. Dynamic exclusion was enabled with a repeat count of 2, repeat duration of 7 s, and exclusion duration of 7 s. Only charge states 2 to 6 were selected for fragmentation. MS2s were generated at top speed for 3 s. Higher-energy collisional dissociation (HCD) was performed on all selected precursor masses with the following parameters: isolation window of 2 m/z, 29% normalized collision energy, Orbitrap detection (resolution of 7,500), maximum inject time of 50 ms, and a standard automatic gain control target. An additional electron transfer dissociation (ETD) fragmentation of the same precursor was triggered if (1) the precursor mass was between m*/z* 300 to 1500 and (2) 3 of 8 *N*-acetylhexosamine (HexNAc) or NeuAc fingerprint ions (126.055, 138.055, 144.07, 168.065, 186.076, 204.086, 274.092, and 292.103) were present at *m/z* ± 0.1 and greater than 5% relative intensity. Two files were collected for each sample: the first collected an ETD scan with supplemental energy (EThcD) while the second method collected a scan without supplemental energy. Both used charge-calibrated ETD reaction times, 100 ms maximum injection time, and standard injection targets. EThcD parameters were as follows: Orbitrap detection (resolution 7,500), calibrated charge-dependent ETD times, 15% nCE for HCD, maximum inject time of 150 ms, and a standard precursor injection target. For the second file, dependent scans were only triggered for precursors below *m/z* 1000, and data were collected in the ion trap using a normal scan rate.

#### Mass spectrometry data analysis

Raw files were searched using Byonic (version 4.5.2, Protein Metrics, Inc.) against the UniProtKB/Swiss-Prot *Homo sapiens* proteome (Query: proteome: up000005640 AND reviewed: true) and a curated mucin database. Briefly, the mucin database consists of nearly 350 proteins from Uniprot’s annotated human proteome predicted to bear the dense *O*-glycosylation characteristic of mucin domains^28^. For all samples, we used the default *O*-glycan database containing 9 common structures. Raw files were first searched against the human proteome and then the curated mucin database. In both cases, files were searched with semi-specific cleavage N-terminal to Ser and Thr and six allowed missed cleavages. Samples treated with trypsin were searched with the same parameters but also allowed cleavage C-terminal to Arg or Lys. Mass tolerance was set to 10 ppm for MS1’s and 20 ppm for MS2’s. Met oxidation was set as a variable modification and carbamidomethyl Cys was set as a fixed modification. From the Byonic search results, glycopeptides were filtered to a score of > 150 and a logprob of > 1.

Proteomics raw data has been deposited in the *PRoteomics IDEntifications Database (PRIDE)*,* accession number PXD080424.*

#### Proteomic data statistical analysis

Mucins were considered true identifications if they ranked higher than the second-highest false-positive protein (reverse hit) in the Byonic search results. For the pie charts shown in Fig. 4, protein intensity values were first extracted from the Byonic output. To account for differences in natural variation between measurements, each intensity was adjusted using a normalization factor. This factor was calculated from the total combined intensity of the top 500 most abundant proteins identified in the same dataset. The normalized intensities were averaged within each triplicate and used to highlight the five mucins with the highest mean signal intensities; the remaining mucins were grouped as “others.” For the bar graphs in Fig. 4I and J, the area under the extracted ion chromatogram (XIC) peak for a representative tryptic peptide from each mucin (MUC2: ^1170^DRPIYEEDLKK¹¹⁸⁰; MUC5B: ²³⁴⁶AAGGAVCEQPLGLECR²³⁶¹; MUC17: ³⁴AVWDGGGCISQGDVLNR⁵⁰; MUC5AC^56^:GATVFPSLR^64^) was normalized to the intensity of a reference peptide from SmE (¹¹⁹RGENEAAFFAR¹²⁹) within the same sample.

### Statistical analysis

Statistical analysis was performed using GraphPad Prism 8.0.2 (GraphPad Software, La Jolla, CA, USA). Differences among conditions (CTR, ZA, ZD) were analysed using one-way analysis of variance (ANOVA) with Tukey’s post hoc test. Adjusted *p*-values were used to denote significance as follows: *p* < 0.05 (*), *p* < 0.01 (**), *p* < 0.001 (***), and not significant (ns). Data were presented as mean + standard deviation (SD) from at least three independent replicates per condition. For mucinome analysis, differences were assessed using one-way ANOVA for each protein (MUC2, MUC5B, and MUC17), assuming normally distributed residuals and homogeneous variances (verified by the Brown-Forsythe test). When the overall ANOVA was significant (*p* < 0.05), pairwise comparisons were conducted using Tukey’s post-hoc multiple comparisons test.

## Supplementary Information

Below is the link to the electronic supplementary material.

Supplementary Material 1

## Data Availability

The raw data of the transcriptome analysis used in the publication have been deposited in NCBI’s Gene Expression Omnibus and are accessible through GEO Series accession number GSE319402. Proteomics raw data of this manuscript has been deposited in the PRoteomics IDEntifications Database (PRIDE), accession number PXD080424.

## Abbreviations

AAL: Aleuria Aurantia lectin
ACN: Acetonitrile
ANOVA: Analysis of variance
B3GNT6: β-1,3-N-acetylglucosaminyltransferase 6
B4GALT5: β-1,4-galactosyltransferase 5
BCA: Bicinchoninic acid
C1galt1: Glycoprotein-N-acetylgalactosamine 3-β-galactosyltransferase 1
COSMC: C1GALT1 specific chaperone 1
CTR: Control
DBA: Dolichos Biflorus agglutinin
DMEM: Dulbecco’s Modified Eagles Medium
DOL-PP: Dolichoyl-pyrophosphate
DPM1: Dolichol-phosphate mannosyltransferase subunit 1
DTT: Dithiothreitol
EDTA: Ethylenediaminetetraacetic acid
ER: Endoplasmic reticulum
ETD: Electron transfer dissociation
ETDhcD: ETD scan with supplemental energy
FABP1: Fatty acid binding protein 1
FCS: Fetal calf serum
FDR: False discovery rate
FITC: Fluorescein isothiocyanate
Fuc: Fucose
FUT: Fucosyltransferase
Gal: Galactose
GalNAc: N-Acetyl-galactosamine
GCNT: Glucosaminyl (N-acetyl) transferase
GlcNAc: N-acetylglucosamine
GO: Gene ontology
GTs: Glycosyltransferases
GUCA2A: Guanylate cyclase activator 2 A
HCD: Higher-energy collisional dissociation
HEPES: 4-(2-hydroxyethyl)-1-piperazineethanesulfonic acid
HexNac: N-acetylhexosamine
IAA: Iodoacetamide
IBD: Inflammatory bowel disease
ICP–MS: Inductively coupled tandem mass spectrometry
IL-33: Interleukin-33
K2C79: Keratin, type II cytoskeletal 79
KO: Knocking-out
LC-MS/MS: Liquid chromatography-tandem mass spectrometry
LSM: laser scanning microscopy
MAL II: Maackia Amurensis lectin II
MALDI-TOF: Matrix-assisted laser desorption/ionization time of flight
Man: Mannose
MAN: Mannosidase alpha class
MAN1A: MAN 1 A member
MAN2B2: MAN 2B member 2
MMP7: Matrix metallopeptidase 7
MOC: Mander’s overlap coefficient
MOGS: Mannosyl-oligosaccharide glucosidase
MS: Mass spectrometry
MWCO: Molecular weight cut off
NC: Nitrocellulose
NeuAC: Sialic acid
OST: Oligosaccharyltransferase
PAGE: Polyacrylamide gel electrophoresis
PBS: Phosphate-buffered saline
PCA: Principal Component Analysis
PGBM: Basement membrane-specific heparan sulfate proteoglycan core protein
PNA: Peanut agglutinin
PODXL: AGRIN, podocalyxin-like protein 1
POFUT1: Protein O-FUT 1
PVDF: Polyvinylidene difluoride
RIPA: Radioimmunoprecipitation assay buffer
RMA: Robust multiarray average
SD: Standard deviation
SDC: Sodium deoxycholate
SDS: Sodium dodecyl sulfate
SLC: Solute carrier
SmE: Serratia marcescens enhancin
SNA: Sambucus Nigra lectin
SPE: Solid-phase extraction
SRD5A3: Steroid 5 -reductase 3
ST3GAL: ST3 β-galactoside α-2,3-sialyltransferase
ST6GALNAC: ST6 N-acetylgalactosaminide α-2,6-sialyltransferase
ST8SIA6: α-N-acetyl-neuraminide α-2,8-sialyltransferase 6
StcEE447D: Secreted protease of C1 esterase inhibitor
TBS-T: Tris-buffered saline with Tween^®^ 20
TE: Trace element
TNF: Tumor necrosis factor
TNFRSF11B: TNF receptor superfamily member 11b
TRITC: Tetramethylrhodamine isothiocyanate
UEA I: Ulex Europaeus agglutinin I
WB: Western blot
WGA: Wheat germ agglutinin
XIC: Extracted ion chromatogram
ZA: Zinc-adequate
ZD: Zinc-deficient
ZIPs: Zn/iron-regulated transporter-like proteins
Zn: Zinc
ZnT: Zn transporter
ZP1: Zinpyr-1
