# Supplementary material for: Transcriptome, glycome, and mucinome analysis reveal zinc is essential for the composition of mucus in the human goblet cell model HT-29-MTX
Source: Sci Rep. 2026 Aug 11;16:24822. doi: 10.1038/s41598-026-66227-4 (PMC13458581; doi:10.1038/s41598-026-66227-4)
Supplement: Supplementary file 1 [file 41598_2026_66227_MOESM1_ESM.pdf]

Supplementary Material: Transcriptome, glycome, and mucinome analysis reveal zinc is essential for the composition of mucus in the human goblet cell model HT-29-MTX

Christoph Schüßler [1], Nara Chung [2], Renaud Léonard [3], Heike Sprenger [4], Tari Rödel [1], Keira E. Mahoney [2], Susanne Thomsen [5], Esther Ocket [6], Claudia Matthaeus [6], Jordan Denis [3], Franziska Ebert [1], Marvin Wolf [5], Willy Morelle [3], François Foulquier [3], Albert Braeuning [4], Catherine Robbe Masselot [3], Stacy A. Malaker [2], Maria Maares [1,*]

[1] Department of Food Chemistry, Institute of Nutritional Science, University of Potsdam, Potsdam-Golm, Germany
[2] Department of Chemistry, Yale University, New Haven, Connecticut, United States
[3] Université de Lille, CNRS, UMR 8576 - UGSF, Lille, France
[4] German Federal Institute for Risk Assessment (BfR), Department Chemical and Product Safety, Berlin, Germany
[5] Department of Food Chemistry and Toxicology, Technische Universität Berlin, Berlin, Germany
[6] Department of Cellular Physiology of Nutrition, Institute of Nutritional Science, University of Potsdam, Potsdam-Golm, Germany

*Corresponding author:
Maria Maares
Department of Food Chemistry, Institute of Nutritional Science, University of Potsdam, Karl-Liebknecht-Str. 24-25, 14476 Potsdam, Germany
E-mail: maria.maares.1@uni-potsdam.de
Phone: +49(0)331 977295508

Keywords: glycosylation/ intestinal mucins/ trace elements/ zinc deficiency

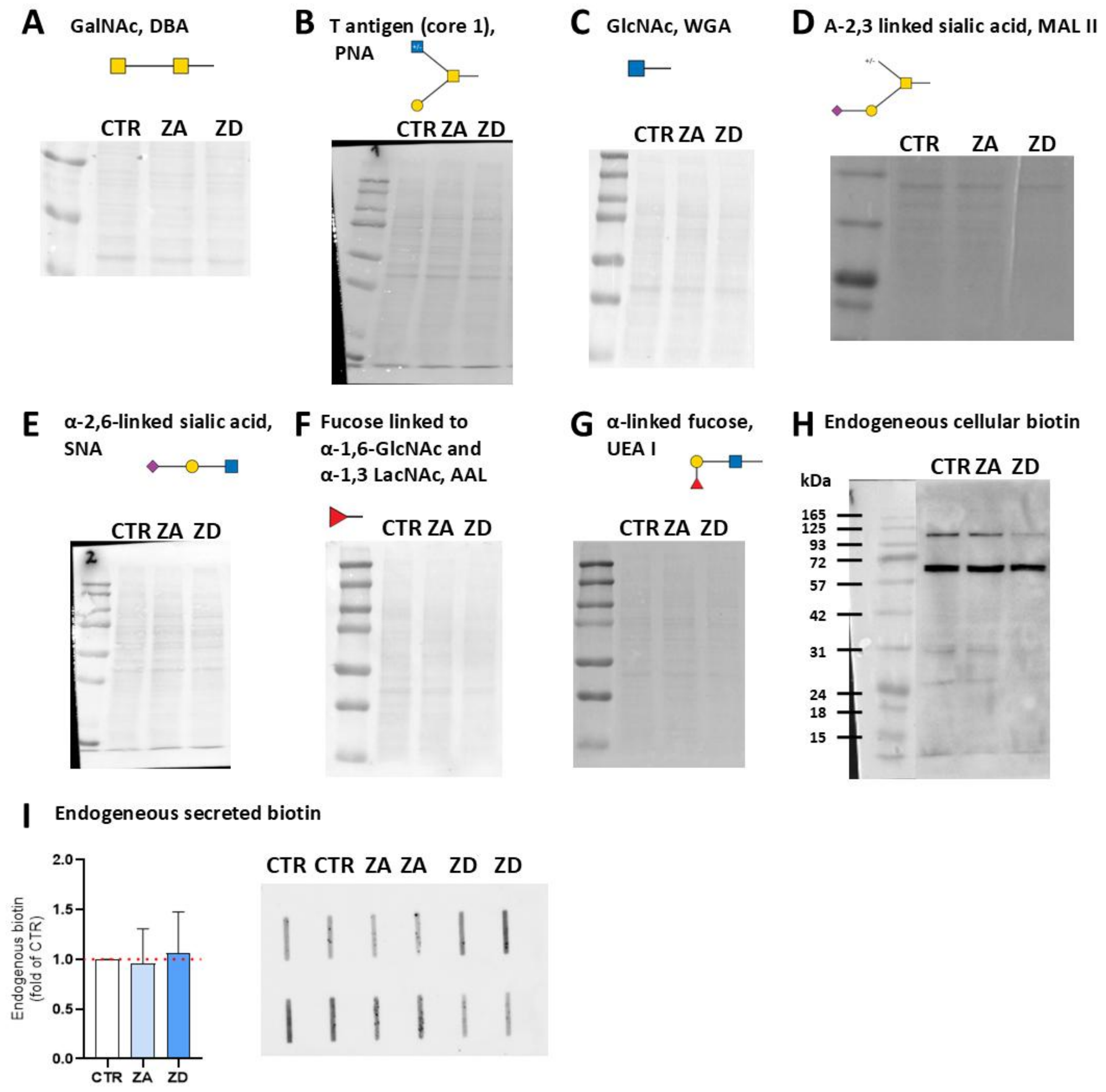

Supplementary Figure 1: Ponceau staining of lectin-blots and endogenous biotin in cells and supernatants of HT-29-MTX cells cultured in CTR, ZA and ZD medium.

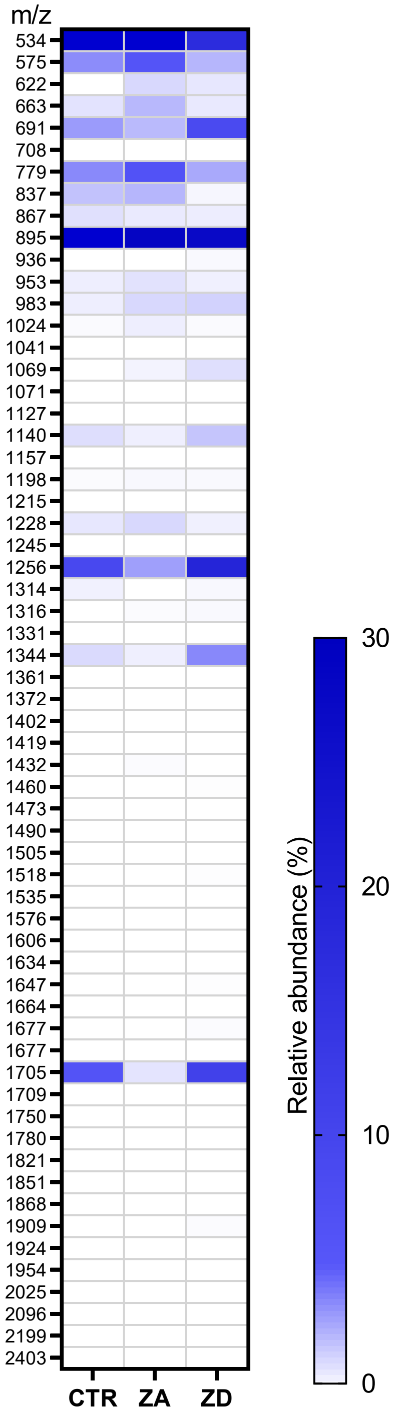

Supplementary Figure 2: Relative abundance of *O*-glycans in HT-29-MTX cells. The relative percentage of each oligosaccharide was calculated based on the integration of peaks on MS spectra. Results are presented as the mean for each oligosaccharide under the same condition.

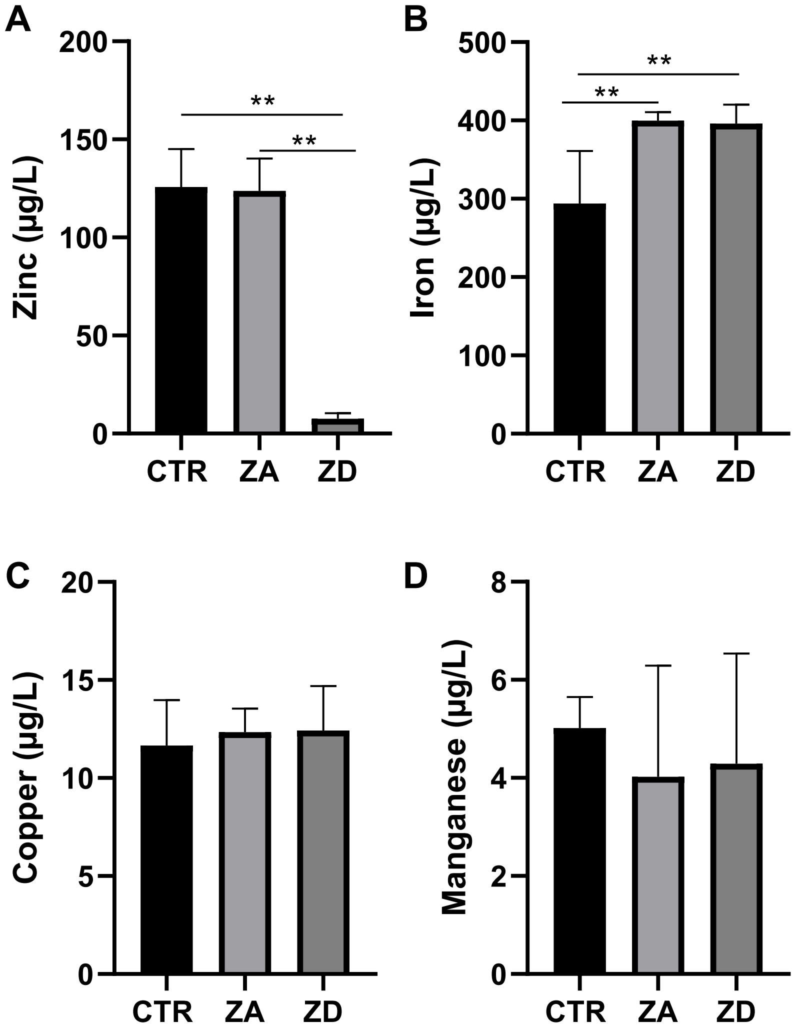

Supplementary Figure 3: Metal concentration of Chelex-treated cell culture media.
Shown are mean + SD of n = 3 replicates.

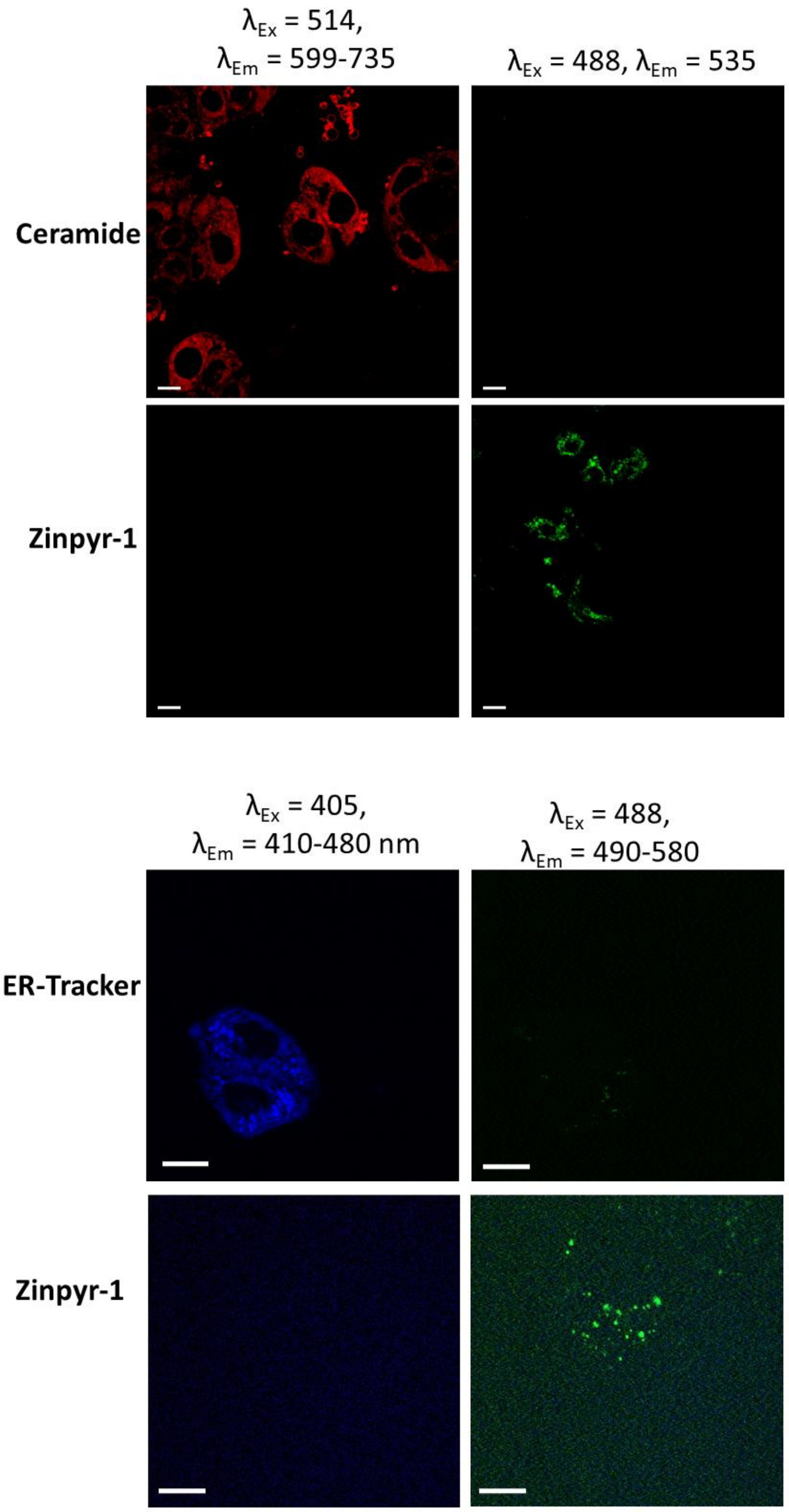

Supplementary Figure 4: Fluorescent channel bleed-through in confocal laser scanning microscopy analysis. HT-29-MTX cells were cultured for 2 days and then incubated with either fluorescently labelled ceramide, to stain the Golgi apparatus, or with the zinc-sensitive probe Zinpyr-1. Images were acquired with excitation and emission settings specific for 2-TR-Ceramide or the ER tracker (ER-Tracker™ Blue-White DPX) and Zinpyr-1, demonstrating the absence of signal bleed-through between the two channels. Scale bar = 10 µm

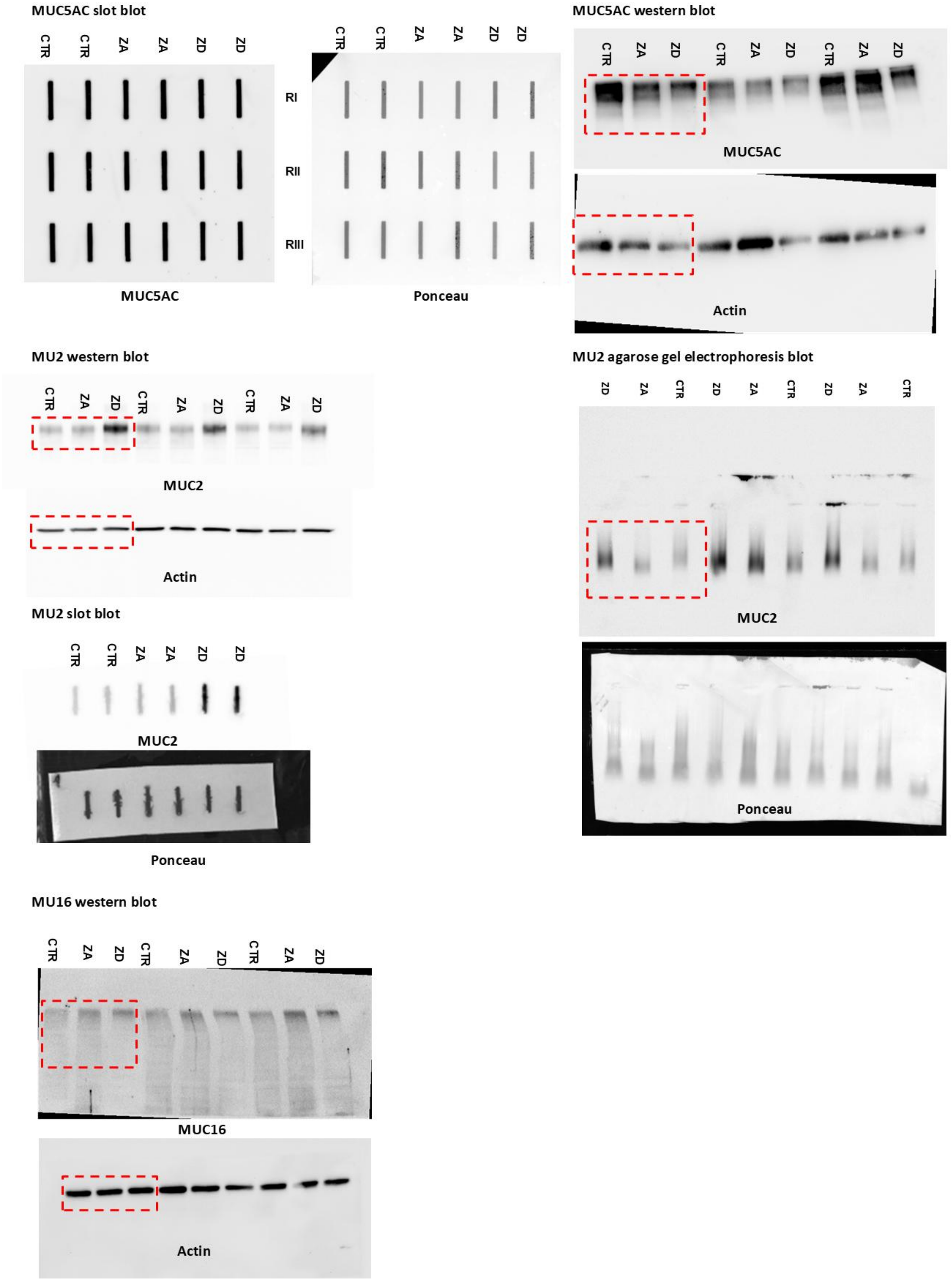

Supplementary Figure 5: Original blots from Figure 4.

Lanes from western blots used for Figure 4K-L, N, and P are outlined in red.

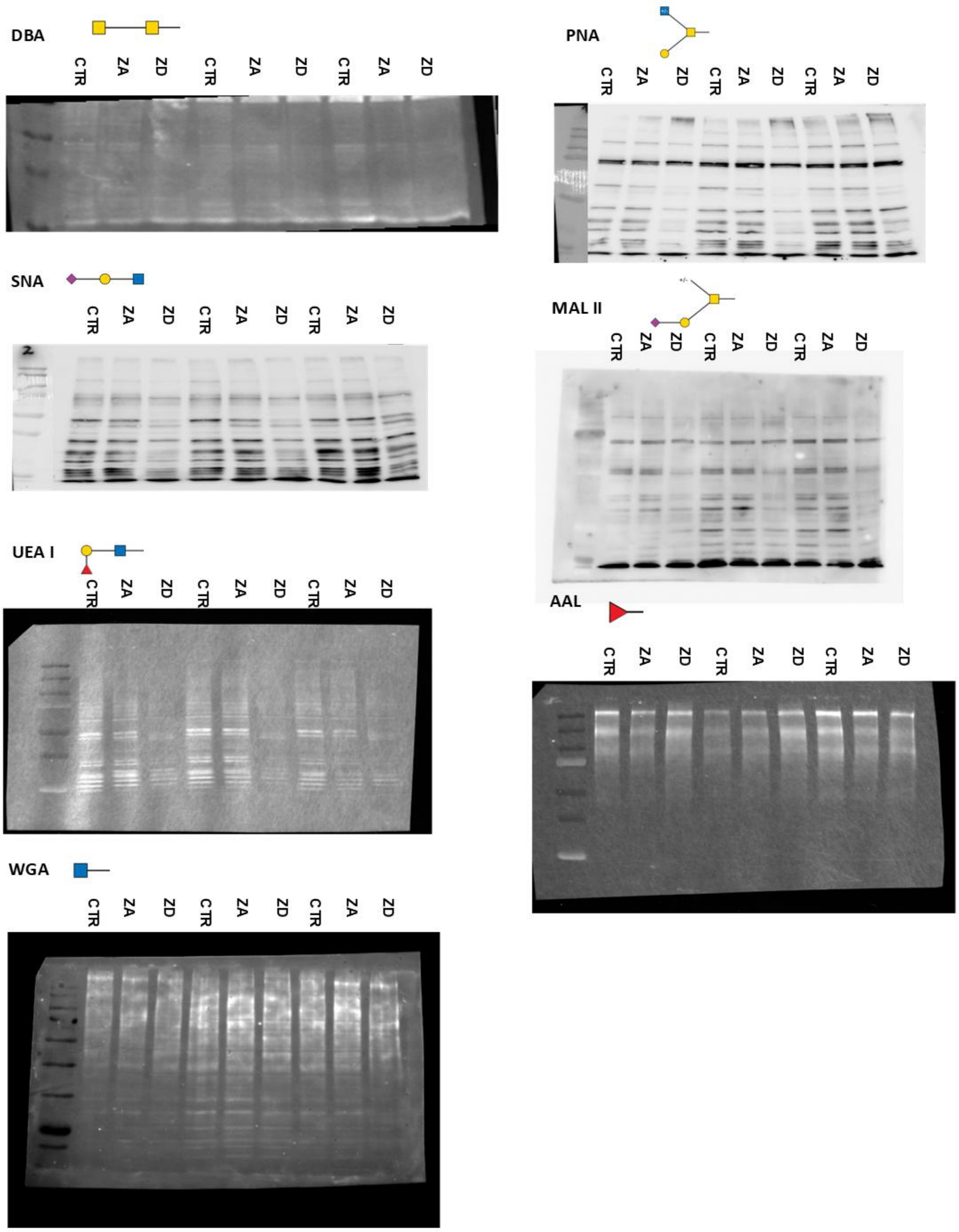

Supplementary Figure 6: Original lectin blots from Figure 5.

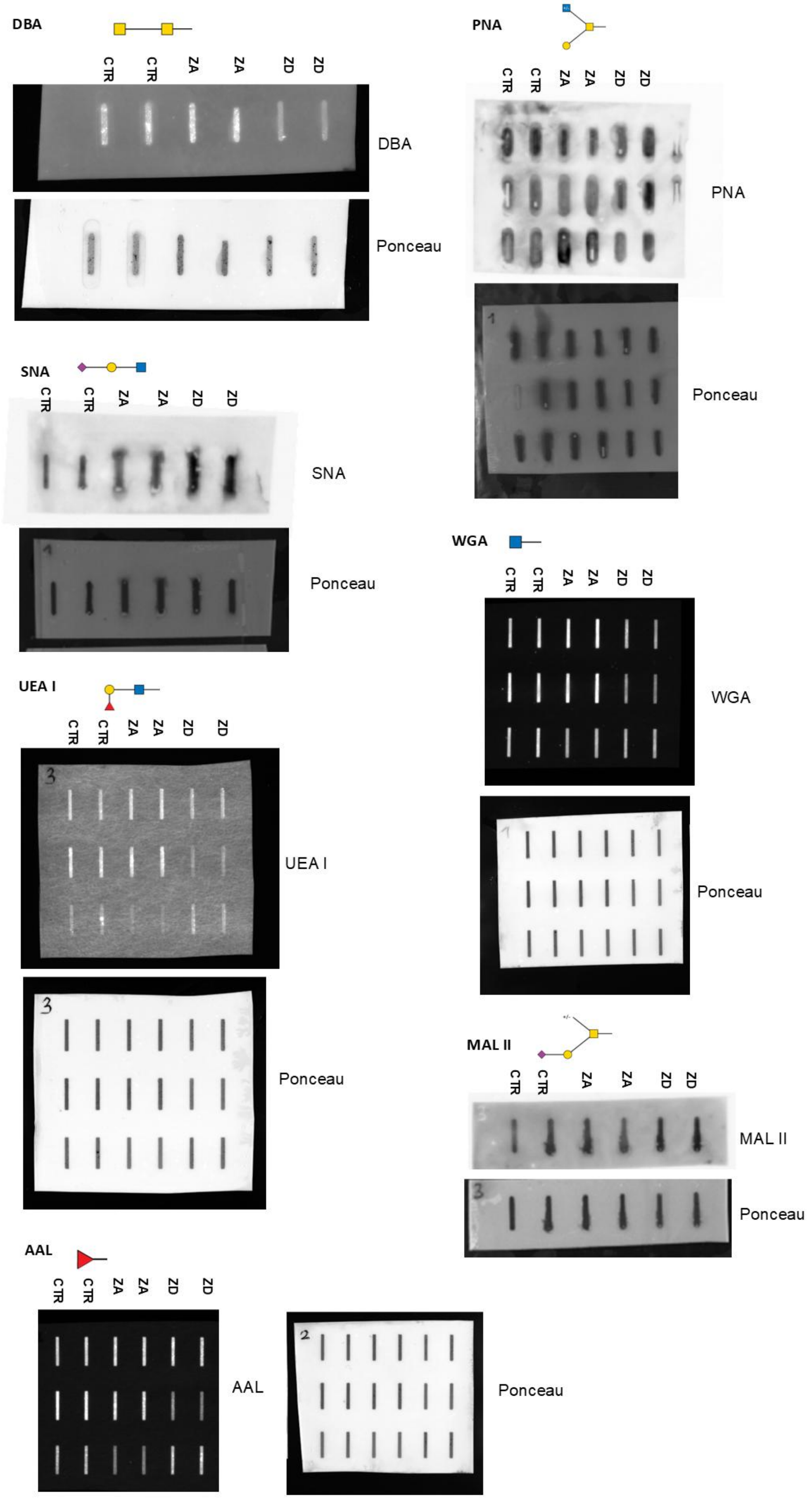

Supplementary Figure 7: Original slot blots from Figure 5.

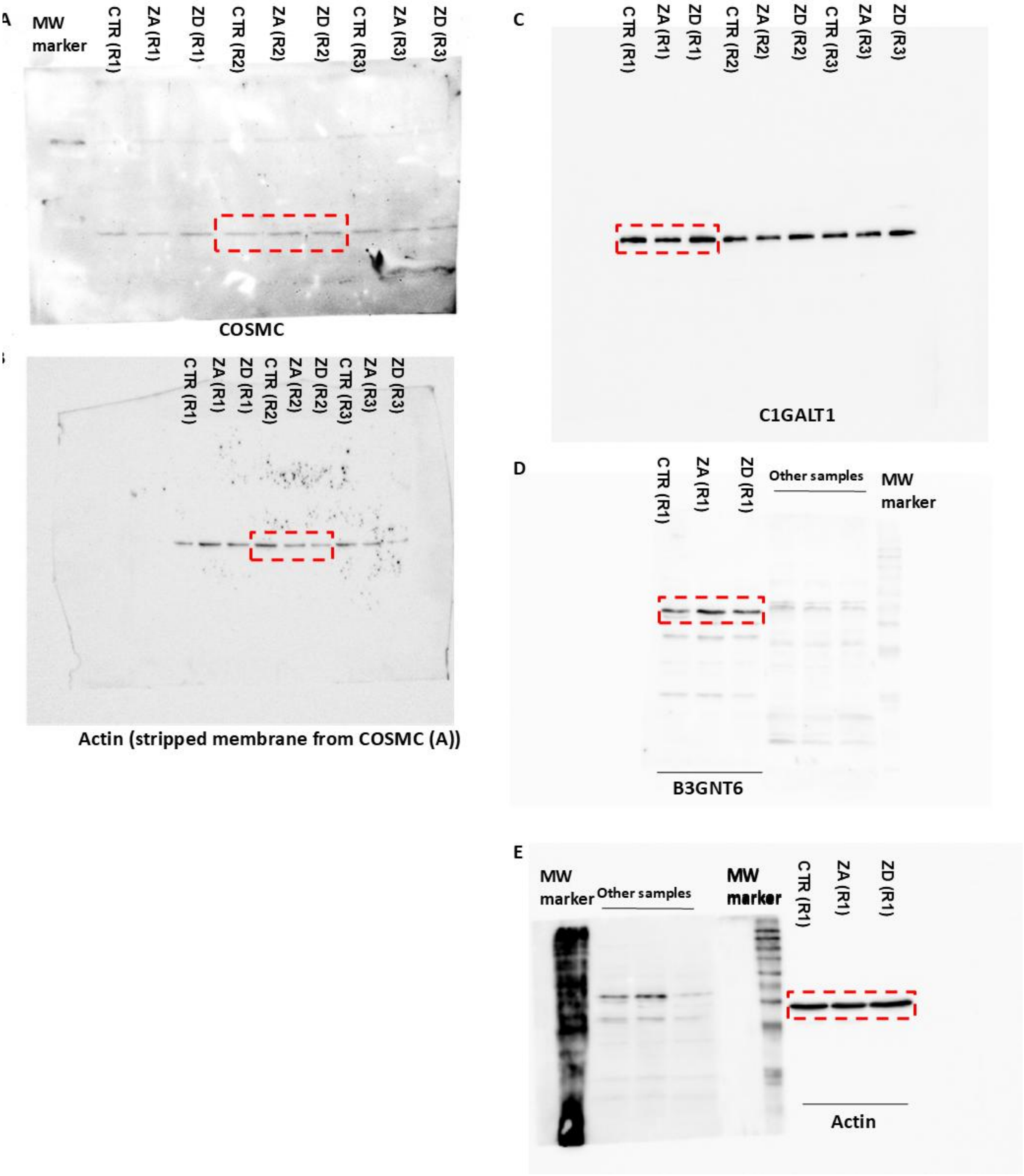

Supplementary Figure 8: Original slot blots from Figure 6E.

As a molecular weight (MW) ladder, a rainbow marker was used, which is not visible in the chemiluminescence channel used to detect the respective proteins. Only in Figure E is a biotinylated molecular weight marker visible. Lanes from western blots used for Figure 6E are outlined in red.

Supplementary Table 1: Proposed neutral and acidic oligosaccharide structures or sequences identified in mucins from HT-29-MTX.

Proposed structures or sequences of oligosaccharides	$[M+Na]^+$	Information
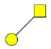	534	T antigen, core 1
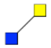	575	Core 3
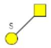	622	Sulfated core 1
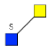	663	Sulfated core 3
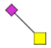	691	Sialyl Tn antigen
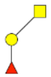	708	Core 1
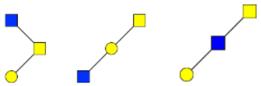	779	Core 1, 2 or 3
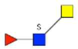	837	Sulfated core 3
1 HexNAc, 1 Hex, 1 SO3, GalNAcol	867	

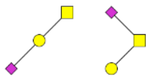	895	Sialylated core 1
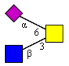	936	Sialylated core 3
1 HexNAc, 1 Hex, 1 Fuc, GalNAcol	953	
1 HexNAc, 2 Hex, 1 GalNAcol	983	
2 HexNAc, 1 Hex, 1 GalNAcol	1024	
1 HexNAc, 1 Hex, 1 Fuc, 1 SO3, GalNAcol	1041	
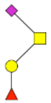	1069	
1 HexNAc, 2 Hex, 1 SO3, GalNAcol	1071	
1 HexNAc, 1 Hex, 2 Fuc, GalNAcol	1127	
1 HexNAc, 1 Hex, 1 NeuAc, GalNAcol	1140	
1 HexNAc, 2 Hex, 1 Fuc, GalNAcol	1157	
2 HexNAc, 1 Hex, 1 Fuc, GalNAcol	1198	
1 HexNAc, 1 Hex, 2 Fuc, 1 SO3, GalNAcol	1215	
2 HexNAc, 2 Hex, GalNAcol	1228	
1 HexNAc, 2 Hex, 1 Fuc, 1 SO3, GalNAcol	1245	

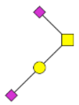	1256	
1 HexNAc, 1 Hex, 1 Fuc, 1 NeuAc, GalNAcol	1314	
2 HexNAc, 2 Hex, 1 SO3, GalNAcol	1316	
1 HexNAc, 2 Hex, 2 Fuc, GalNAcol	1331	
1 HexNAc, 2 Hex, 1 NeuAc, GalNAcol	1344	
3 Hex, 1 HexNAc, 1 Fuc, GalNAcol	1361	
2 HexNAc, 1 Hex, 2 Fuc, GalNAcol	1372	
2 HexNAc, 1 Hex, 1 NeuAc, GalNAcol	1385	
2 HexNAc, 2 Hex, 1 Fuc, GalNAcol	1402	
1 HexNAc, 2 Hex, 2 Fuc, 1 SO3, GalNAcol	1419	
2 HexNAc, 3 Hex, GalNAcol	1432	
2 HexNAc, 1 Hex, 2 Fuc, 1 SO3, GalNAcol	1460	
3 HexNAc, 2 Hex, GalNAcol	1473	
2 HexNAc, 2 Hex, 1 Fuc, 1 SO3, GalNAcol	1490	
1 HexNAc, 2 Hex, 3 Fuc, GalNAcol	1505	
1 HexNAc, 2 Hex, 1 Fuc, 1 NeuAc, GalNAcol	1518	

1 HexNAc, 3 Hex, 2 Fuc, GalNAcol	1535	
2 HexNAc, 2 Hex, 2 Fuc, GalNAcol	1576	
2 HexNAc, 3 Hex, 1 Fuc, GalNAcol	1606	
2 HexNAc, 1 Hex, 3 Fuc, 1 SO3, GalNAcol	1634	
3 HexNAc, 2 Hex, 1 Fuc, GalNAcol	1647	
2 HexNAc, 2 Hex, 2 Fuc, 1 SO3, GalNAcol	1664	
3 HexNAc, 3 Hex, GalNAcol	1677	
1 HexNAc, 2 Hex, 2 NeuAc, GalNAcol	1705	
1 HexNAc, 3 Hex, 3 Fuc, GalNAcol	1709	
2 HexNAc, 2 Hex, 3 Fuc, GalNAcol	1750	
2 HexNAc, 3 Hex, 2 Fuc, GalNAcol	1780	
3 HexNAc, 2 Hex, 2 Fuc, GalNAcol	1821	
3 HexNAc, 3 Hex, 1 Fuc, GalNAcol	1851	
2 HexNAc, 3 Hex, 2 Fuc, 1 SO3, GalNAcol	1868	
3 HexNAc, 2 Hex, 2 Fuc, 1 SO3, GalNAcol	1909	
2 HexNAc, 2 Hex, 4 Fuc, GalNAcol	1924	
2 HexNAc, 3 Hex, 3 Fuc, GalNAcol	1954	
3 HexNAc, 3 Hex, 2 Fuc, GalNAcol	2025	
4 HexNAc, 3 Hex, 1 Fuc, GalNAcol	2096	

3 HexNAc, 3 Hex, 3 Fuc, GalNAcol	2199	
3 HexNAc, 4 Hex, 3 Fuc, GalNAcol	2403	
